# Inflammatory bowel diseases: pathological mechanisms and therapeutic perspectives

**DOI:** 10.1186/s43556-025-00395-z

**Published:** 2026-01-07

**Authors:** Xiaona Yang, Hong Guo, Min Zou

**Affiliations:** 1 Chongqing Medical University, Chongqing, 400016 China; 2 Department of Gastroenterology, Chongqing General Hospital, Chongqing University, Chongqing, 401147 China

**Keywords:** IBD, CD, UC, Pathophysiology, Therapy

## Abstract

Inflammatory bowel disease (IBD) is a heterogeneous group of disorders characterized primarily by chronic relapsing intestinal inflammation, encompassing Crohn’s disease (CD) and ulcerative colitis (UC), affecting individuals across age groups with variable clinical manifestations. With the advancement of global industrialization, its incidence continues to rise, particularly in newly industrialized regions, which not only severely impairs patients' quality of life but also emerges as a major public health concern threatening digestive system health, accompanied by a substantial healthcare burden, thus necessitating the development of more effective and safer individualized treatment strategies. This review summarizes the pathogenesis of IBD, including intestinal mucosal immune dysregulation, intestinal barrier damage, gut microbiota dysbiosis, reactive oxygen species (ROS) homeostasis imbalance, and the complex crosstalk between genetic and environmental factors; however, clinical treatment still faces numerous challenges: 30%-40% of patients exhibit primary or secondary non-response to existing therapeutic regimens such as biologics and small-molecule drugs, and prolonged administration tends to induce significant side effects. Further integrated herein are emerging strategies such as ROS modulators, novel immune-targeted modulation, intestinal barrier repair agents, microbiota-directed interventions, multi-omics-based precision medicine, and artificial intelligence (AI)-assisted therapy, which represent key directions to address the limitations of traditional treatments. This article begins with an overview of basic pathological mechanisms and offers a comprehensive overview of relevant therapeutic approaches and future development directions, aiming to facilitate the transition of the field from traditional generalized therapies to personalized precision medicine and to bridge the long-standing gap between basic research and clinical practice.

## Introduction

IBD is a heterogeneous group of disorders characterized by chronic-relapsing intestinal inflammation and is predominantly subclassified into CD and UC. During the past decade, both the incidence and prevalence of IBD have increased in numerous countries, notably in Western and newly industrialized nations [1, 2]. According to the 2017 Global Burden of Disease (GBD) study, the worldwide number of IBD cases reached 6.8 million, and the age-standardized prevalence increased from 79.5 per 100,000 persons in 1990 to 84.3 per 100,000 in 2017 [3]. In North America, the prevalence was estimated at 725 per 100,000 persons in 2018 and is projected to reach 981 per 100,000 by 2030 [4]. Although UC remains the predominant phenotype across Asia, the UC-to-CD prevalence ratio has progressively narrowed in several regions [5]. Importantly, the age at onset has shifted downward, with the majority of patients developing symptoms during childhood or adolescence [6]. IBD is frequently complicated by intestinal perforation, stricture formation, and colorectal neoplasia, thereby imposing substantial long-term morbidity, impairing quality of life, and amplifying the global socioeconomic and health-care burden.

Advances in molecular biology, immunology, and microbiome technologies have enabled substantial breakthroughs in IBD pathogenesis research. At the immunological level, a central role has been established for the imbalance between T helper 1 cell (Th1)/T helper 17 cell (Th17) and Regulatory T cell(Treg cell) subsets [7] and for excessive activation of innate immune receptors [8]. From the microbial perspective, reduced diversity of the gut microbiota and the depletion of beneficial taxa have been causally linked to disease progression. At the barrier level, aberrant intercellular junctional proteins (tight-junction, adherens-junction, and cytoskeleton-associated proteins) [9] and increased programmed cell death of intestinal epithelial cells [10] have been identified as key pathogenic mechanisms. In addition, oxidative stress triggered by disruption of redox homeostasis has been recognized as a critical driver of mucosal injury and inflammatory amplification [11, 12]. Gene–environment interactions involving susceptibility loci [13, 14] and environmental exposures [15, 16] have been increasingly clarified. Nevertheless, the current literature remains highly fragmented, with most investigations focusing on a single pathogenic module, while the synergistic regulatory networks among pathways have not been systematically characterized. This knowledge gap has markedly impeded the identification and translation of precision therapeutic targets, thereby constituting the primary motivation for the present review.

Currently, the mainstream therapeutic armamentarium for IBD encompasses conventional agents (5-aminosalicylates, corticosteroids, and traditional immunosuppressants such as azathioprine), biologics, and small-molecule inhibitors, all of which have demonstrated efficacy in inducing and maintaining remission and in ameliorating symptomatic burden. Nevertheless, the limited targeting capacity of conventional drugs results in suboptimal drug concentrations at sites of active intestinal inflammation, thereby restricting therapeutic effectiveness. Owing to the pronounced heterogeneity of IBD, biologics and small-molecule agents elicit highly variable responses, with approximately 30–40% of patients experiencing primary or secondary loss of response [17]. Concurrently, the substantially higher health-care expenditures associated with these therapies constitute an additional barrier to their widespread adoption. Consequently, emerging therapeutic strategies that directly target the core pathophysiological mechanisms of IBD have been developed, offering a new avenue to overcome the limitations of existing regimens and to improve long-term outcomes.

This review first systematically delineates the core pathophysiological mechanisms of IBD and clarifies the inter-regulatory relationships among individual pathogenic modules. Next, the clinical landscape and principal limitations of current therapeutics are summarized, enabling the identification of critical unresolved issues. Subsequently, emerging mechanism-based therapeutic strategies are highlighted, and their translational progress and clinical potential are critically appraised. Finally, by analyzing the translational gap between conventional and emerging approaches, the imperative for precision, mechanism-oriented individualized therapy is emphasized, thereby providing a comprehensive reference for both fundamental research and clinical practice in the field.

## Pathophysiology of IBD

**Fig. 1:**
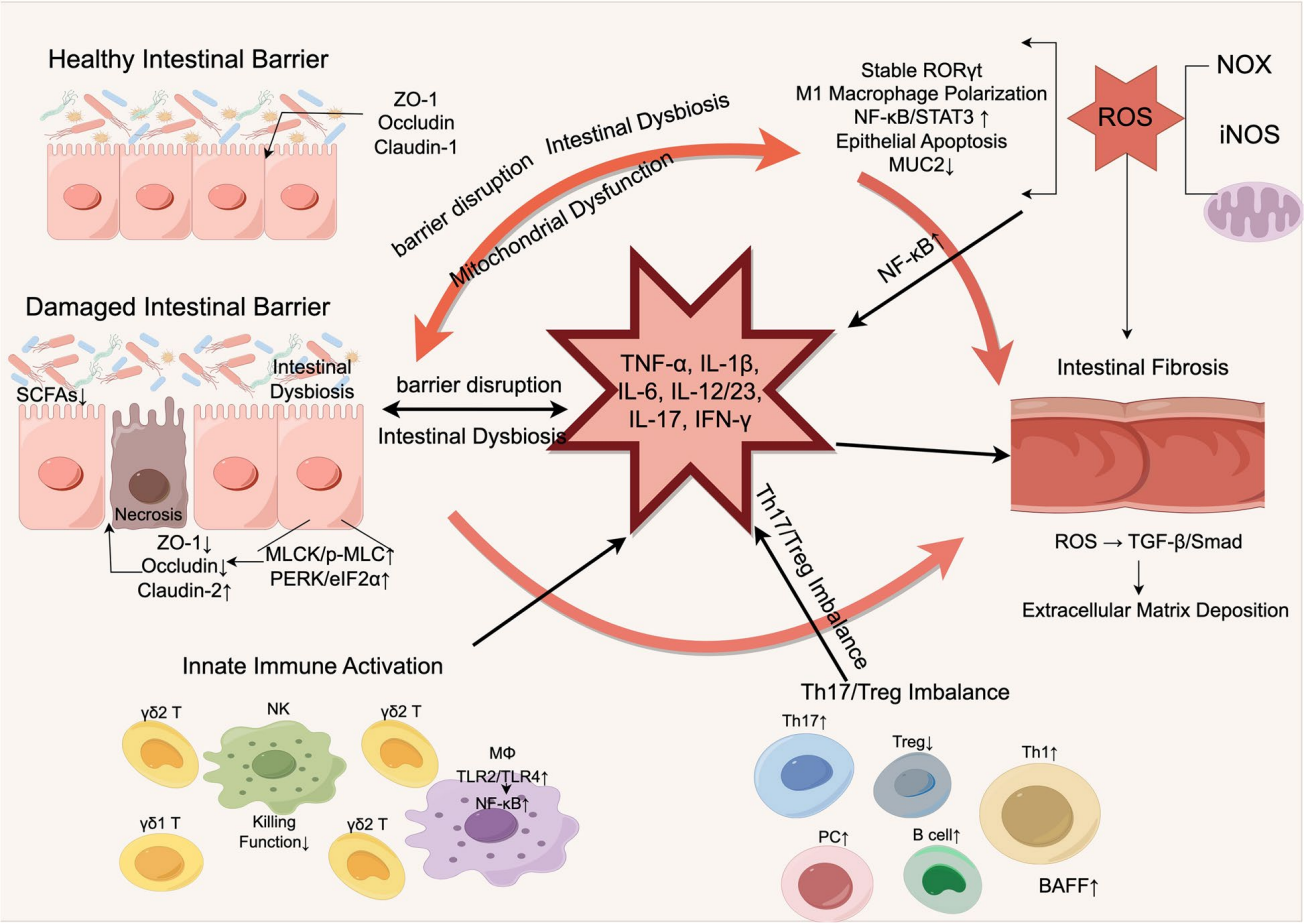
Core pathogenic circuitry in inflammatory bowel disease. In the healthy state, the epithelial barrier is sealed by high ZO-1, Occludin and Claudin-1, crowned by a dense mucus layer rich in short-chain fatty acid–producing commensals. Disease begins with barrier disintegration: tight-junction proteins are down-regulated, Claudin-2 is aberrantly up-regulated, MLCK and PERK/eIF2α stress pathways are activated, and epithelial apoptosis increases while butyrate-generating microbiota decline and pathobionts expand. Subjacent lamina-propria macrophages sense translocating microbes via TLR2/4, triggering NF-κB–dependent release of TNF-α, IL-1β, IL-6 and IL-23. NK and γδ-T cells lose cytotoxic competence yet secrete IL-17. Th1 and Th17 lineages expand, Treg cells are functionally silenced, and BAFF-stimulated plasma cells accumulate, generating a cytokine storm. NADPH oxidase, inducible nitric-oxide synthase and dysfunctional mitochondria jointly produce reactive oxygen species that stabilize RORγt, sustain M1 macrophage polarization, activate STAT3, deplete MUC2, and, via TGF-β/Smad signalling, drive extracellular-matrix deposition and intestinal fibrosis. Oxidative injury and persistent inflammation feed back onto the epithelium, perpetuating a self-reinforcing pathogenic cycle. Note: ↑: Increased; ↓: Decreased. ZO-1: Zonula Occludens-1; SCFAs: Short-Chain Fatty Acids; MLCK/p-MLC: Myosin Light Chain Kinase/Phosphorylated Myosin Light Chain; PERK/eIF2α: Protein Kinase R-like Endoplasmic Reticulum Kinase/Phosphorylated Eukaryotic Initiation Factor 2α; TNF-α: Tumor Necrosis Factor-α; IL: Interleukin; IFN-γ: Interferon-γ; γδ1 T: Gamma delta 1 T cell; γδ2 T: Gamma delta 2 T cell; NK: Natural Killer cell; MΦ: Macrophage; TLR: Toll-Like Receptor; NF-κB: Nuclear Factor κ-Light-Chain-Enhancer of Activated B Cells; Th: T Helper cell; Treg: Regulatory T cell; PC: Plasma Cell; BAFF: B Cell Activating Factor; RORγt: RAR-Related Orphan Receptor γt; STAT3: Signal Transducer and Activator of Transcription 3; MUC2: Mucin 2; ROS: Reactive Oxygen Species; NOX: NADPH Oxidase; iNOS: Inducible Nitric Oxide Synthase; TGF-β: Transforming Growth Factor-β; Smad: Mothers Against Decapentaplegic Homolog proteins. (Figure was created by figdraw.com)

A comprehensive understanding of the pathophysiological mechanisms underlying IBD is considered fundamental to the development of novel precision therapeutic strategies. It has been extensively documented that persistent intestinal mucosal inflammation and tissue damage constitute the core pathological manifestations of IBD, which are driven by dysfunctions in multiple physiological regulatory systems: At the immunological level, aberrant activation of both innate and adaptive immune responses, in conjunction with cytokine network dysregulation, has been shown to directly drive the initiation and amplification of inflammatory responses [18–20]; At the barrier level, increased apoptosis of intestinal epithelial cells [21], aberrant expression of tight junction proteins [22], and endoplasmic reticulum stress-mediated functional impairments [23] have been demonstrated to compromise the intestinal “first line of defense”; At the microbiological level, structural remodeling and functional abnormalities of the intestinal microbiota have been identified as key triggers for both initiation and maintenance of inflammation through metabolic interactions and immune regulatory pathways [24–28]; At the oxidative stress level, excessive generation of ROS and antioxidant system imbalance have been observed to not only directly damage intestinal mucosal cells but also to influence immune cell differentiation and barrier repair through modulation of signaling pathways, thereby creating a vicious cycle of inflammatory progression [12, 29]. Herein, these four key mechanisms are systematically examined with respect to their molecular regulatory pathways, cellular biological characteristics, and clinical associations, with the aim of providing a comprehensive reference for mechanistic research and therapeutic target development in IBD (Fig. 1).

### Immune dysregulation in the intestinal mucosa

Immune dysregulation in the intestinal mucosa is characterized by the abnormal activation of innate immunity and the excessive response of adaptive immunity, particularly the imbalance of pro-inflammatory cytokines such as tumor necrosis factor-alpha (TNF-α). This dysregulation is directly associated with disease activity and clinical manifestations.

Innate immune dysregulation plays an important role in the pathogenesis of IBD. Several mechanisms are associated with the pathophysiology of IBD. Toll-like receptors (TLRs) are important pattern recognition receptors in the innate immune system, responsible for recognizing microbial components and initiating immune responses. Studies have shown that the expression of TLR2, TLR4, and their co-receptor cluster of differentiation 14 (CD14) is significantly upregulated in the intestinal mucosa of IBD patients, particularly in active UC and CD cases [8].This upregulation may lead to an excessive recognition of gut microbiota, triggering abnormal inflammatory responses. For example, TLR4 activates the nuclear factor kappa-light-chain-enhancer of activated B cells (NF-κB) signaling pathway through the recognition of lipopolysaccharides (LPS), promoting the release of pro-inflammatory cytokines and exacerbating intestinal inflammation [8]. Moreover, the expression patterns of TLR2 and TLR4 vary across different subtypes and disease stages of IBD, suggesting that they may play distinct roles at different stages of the disease [8, 30]. The TLR7 agonist Imiquimod alleviates DSS-induced colitis in mice by inducing the expression of type I interferons (IFN) and antimicrobial peptides (AMPs) and inhibits the survival of intracellular pathogens such as Salmonella [31]. However, abnormalities in pattern recognition receptor(PRR) signaling may also contribute to the pathogenesis of IBD. For instance, certain PRR-deficient mice exhibit varying susceptibility to colitis, indicating the complex roles of PRRs in regulating microbiota composition, epithelial repair, and immune cell activation [32].

The oxygen gradient in the intestinal mucosa is crucial for maintaining intestinal homeostasis, while IBD patients often exhibit significant mucosal hypoxia. Hypoxia-inducible factor 1-alpha (HIF-1α) and hypoxia-inducible factor 1-alpha (HIF-2α) are key transcription factors in the hypoxic signaling pathway, but their roles in IBD are dualistic. There are significant differences in the expression and functions of HIF-1α and HIF-2α in IBD: HIF-1α exerts anti-inflammatory and protective effects by upregulating the expression of barrier-protective genes and antimicrobial peptides, whereas HIF-2α promotes epithelial cell-mediated inflammatory responses and proliferation, which are associated with chronic inflammation and carcinogenesis [33]. In IBD patients, the degree of mucosal hypoxia exacerbates, and the activation of hypoxia-inducible factors (HIFs) may be a critical factor in disease progression.

NADPH oxidase (NOX) and dual oxidase (DUOX) are the main sources of ROS in the gut, and the ROS they produce play an important role in maintaining intestinal homeostasis and antimicrobial defense. However, in IBD patients, the abnormal expression of NOX1 and DUOX2 may lead to excessive ROS production, exacerbating oxidative stress and intestinal damage [33]. ROS derived from epithelial cells (NOX1/DUOX2) and phagocytes (NOX2) exert contradictory roles in intestinal homeostasis. Under physiological conditions, ROS maintain mucosal defense by regulating bacterial virulence, epithelial repair, and microbial communities; however, excessive ROS generation accelerates IBD progression [34]. Notably, the antimicrobial function of DUOX2 is highly conserved in evolution, and its gene mutations are associated with early-onset IBD. Treatment targeting NOX/DUOX requires precise balance: inhibitors may be used for high ROS states, while inducers may be suitable for patients with functional deficiencies [34].

Natural killer (NK) cells and γδ T cells are essential components of the innate immune system in the intestinal mucosa. Circulating NK cells in IBD patients exhibit distinct metabolic dysregulation, including reduced mitochondrial mass, impaired oxidative phosphorylation capacity, and mechanistic target of rapamycin complex 1 (mTORC1) signaling inhibition [35]. Despite secreting substantial amounts of Interleukin-17 (IL-17) and TNF-α, these cells show a significant reduction in cytotoxic function, which may lead to an increased risk of infection and carcinogenesis [35]. This finding highlights the importance of immune metabolic reprogramming in IBD pathogenesis and offers a new approach for restoring immune balance by regulating NK cell metabolism. Similarly, gamma delta T (γδ T) cells in the intestinal mucosa of IBD patients undergo subset redistribution: γδ1 T cells are reduced while γδ2 T cells increase, with the latter exhibiting an effector memory phenotype and elevated expression of TNF-α and IL-17, which correlates with disease severity [36]. This phenotypic shift may be associated with the chronic inflammatory state of IBD, particularly as γδ2 T cells, through IL-17 secretion, contribute to the maintenance and exacerbation of inflammation [36]. Furthermore, metabolic abnormalities in γδ T cells, such as mitochondrial dysfunction and decreased mTORC1 activity, further exacerbate their functional impairment, potentially leading to a diminished ability to clear pathogens in IBD patients [36].

Based on the understanding of innate immune dysregulation mechanisms, several studies have explored novel therapeutic approaches targeting TLRs, HIFs, and ROS pathways. For instance, the TLR7 agonist Imiquimod significantly alleviates dextran sodium sulfate (DSS)-induced colitis in mice by inducing the expression of type IFN and antimicrobial peptides [31]. Similarly, immune-stimulating DNA sequences (ISS-DNA) suppress colonic inflammation by activating the innate immune response and show therapeutic potential in various IBD models [37]. Moreover, nanodrugs such as copper sulfide (CuS)/disulfiram (DSF)/Exosome-like (EL)/polyvinylpyrrolidone (PVP) exert therapeutic effects on colitis by modulating C-type lectin receptor (CLR) signaling and the gut microbiota [38]. However, therapeutic strategies such as TLR4 antagonistic antibodies may suppress inflammation but interfere with mucosal repair, suggesting the need to weigh the benefits and risks of treatment [8].

#### Adaptive immune responses in the intestinal mucosa in IBD

In recent years, studies have shown that adaptive immune responses play a key role in the pathogenesis and progression of IBD, particularly the abnormal activation of T cell subsets and cytokine imbalance.

T cells in the intestinal mucosa of IBD patients exhibit marked abnormalities in maturation and differentiation. In treatment-naive IBD patients at the time of diagnosis, researchers identified four distinct T cell maturation profiles based on CD45RA and CD27 expression, with the A profile, enriched with CD45RA + CD27 + naïve T cells, being observed exclusively in the ileum/colon of IBD patients and correlating with increased TNF-α expression and reduced IFN-γ expression [39]. Notably, the expression of inducible costimulatory molecules (ICOS) on CD4 + T cells is significantly increased in the inflammatory mucosa of IBD patients, and this overexpression promotes the production of inflammatory cytokines such as IL-5 in UC and IFN-γ in CD, suggesting that ICOS may serve as a potential therapeutic target for IBD [40].

The distribution and function of Th cell subsets in the intestinal mucosa of IBD patients are significantly altered. Studies have found that Th1 and Th17 cells are significantly increased in the inflammatory mucosa of IBD patients, while the number and function of Treg are suppressed [7]. This imbalance results in the overproduction of pro-inflammatory cytokines, further exacerbating mucosal damage [7, 41]. Simultaneously, the immunosuppressive function of Treg cells is impaired, preventing effective control of the inflammatory response [42]. This imbalance exists not only in CD4 + T cells, but also in CD8 + T cells, which are involved in the inflammatory process, particularly in intraepithelial lymphocytes [43]. Research has shown that enhancing Treg function or inhibiting effector T cell responses can effectively control experimental colitis, thereby validating the central role of this imbalance [42].

In the Th17/Treg balance, Th17 cell polarization is a key driver of inflammation. Studies have found that IL-21 promotes the differentiation of CD4 + T cells into Th17 cells in IBD patients, as indicated by increased expression of IL-17A and retinoid-related orphan receptor gamma t (RORγt) [44]. Simultaneously, mucosal-associated invariant T (MAIT) cells in IBD patients also exhibit a shift toward an IL-17-dominated pro-inflammatory phenotype, displaying IL-17-driven inflammatory characteristics [45, 46]. Single-cell sequencing further revealed a significant increase in HLA-DR + CD38 + T cells in the colonic mucosa of IBD patients, including Treg cells producing inflammatory cytokines [47]. Notably, UC patients characteristically show an increase in IL17A + CD161 + effector memory T cells and IL17A + Treg cells, while CD patients primarily exhibit an increase in IL1B + HLA-DR + CD38 + T cells [47].

The role of B cells in IBD has received increasing attention. Studies have shown that B cells in the intestinal mucosa of IBD patients are significantly activated, and the expression of B cell activating factor (BAFF) is increased in both serum and intestinal tissues. Additionally, fecal BAFF levels have been found to distinguish active IBD patients from healthy controls with 90% sensitivity and 96% specificity [48]. In the unaffected colonic mucosa of colorectal cancer patients, although the frequencies of γδ T cells and tissue-resident memory T cells are decreased, the B cell memory response to commensal bacteria is significantly enhanced, accompanied by an increased proportion of effector memory B cells, transitional B cells, and plasma cells in peripheral blood [49]. Furthermore, it has been found that mesenteric lymph node B cells (MLB) migrate to the intestine in 2,4,6-Trinitrobenzenesulfonic acid (TNBS)-induced colitis through the C–C Chemokine Receptor 8—C–C Chemokine Ligand 1 (CCR8-CCL1)axis and exacerbate the inflammatory response by modulating T cells [50]. These findings suggest that the abnormal activation and migration of B cells are important driving factors for the persistence of inflammation in IBD.

T cells and B cells form a complex network of interactions in the intestinal mucosa. It has been found that MLB can migrate to the intestine and exacerbate colitis by modulating intestinal T cells. These B cells are recruited to the colon by intestinal T cells through the CCR8-CCL1 axis and exacerbate the inflammatory response by enhancing differentiation [50]. In the Senescence-accelerated mouse P1/Yit strain (SAMP1/Yit)mouse model, B cells are involved not only in the development of ileitis but also play a role in the onset of gastritis [51]. Additionally, the interaction between follicular helper T cells and B cells plays a crucial role in the formation of tertiary lymphoid structures, which promote local antibody responses at sites of chronic inflammation [52].

The interaction between innate immunity and adaptive immunity. The innate immune system regulates the initiation and polarization of adaptive immune responses through antigen presentation and cytokine production [53]. Dendritic cells (DCs), as specialized antigen-presenting cells, play a crucial role in determining T cell differentiation fate [54]. Intestinal DCs are capable of inducing protective immunity or immune tolerance, depending on local microenvironmental signals [54]. Additionally, innate lymphoid cells (ILCs), as integral components of the innate immune system, provide early defense before the initiation of adaptive immune responses by producing Th cell-related cytokines, thereby influencing subsequent T cell responses [55, 56].

Several studies have confirmed that the characteristic pathological changes of IBD are due to an imbalance between pro-inflammatory and anti-inflammatory cytokines [57, 58]. In patients with active IBD, pro-inflammatory cytokines such as TNF-α, IL-1β, IL-6, IL-12, and IL-23 are significantly elevated, while anti-inflammatory cytokines such as IL-10 and IL-4 are expressed at insufficient levels [59, 60]. This imbalance not only drives the inflammatory process but also hinders tissue repair and inflammation resolution [57, 58].

The differentiation state of Th cells determines the characteristics of the cytokine profile. It is traditionally believed that CD is primarily associated with a Th1-type response, while UC tends toward a Th2-type response; however, recent studies have found that Th17 cells and their characteristic cytokine IL-17 also contribute to the pathogenesis of IBD [61, 62]. Impaired Treg function further exacerbates the inflammatory response.

In addition to classic cytokines, recent studies have found that members of the IL-1 family(such as IL-36) and the IL-12 family play a crucial role in the pathogenesis of IBD [63, 64]. IL-36 includes pro-inflammatory subtypes and anti-inflammatory subtypes, and its imbalance is associated with various inflammatory diseases [64]. IL-38, a novel member of the IL-1 family, exerts anti-inflammatory effects by binding to receptors such as IL-36R and is abnormally expressed in IBD [65]. These findings provide new targets for the treatment of IBD.

The production of cytokines is regulated by multiple signaling pathways. The nuclear NF-κB pathway is a key regulator of pro-inflammatory cytokine production [66–68]. The melanocortin system antagonizes colonic inflammation by interfering with cytokine imbalance. Sphingosine-1-phosphate (S1P) receptor modulators influence lymphocyte trafficking and cytokine production [69]. Peroxisome proliferator-activated receptor(PPAR) agonists reduce pro-inflammatory cytokine production by inhibiting NF-κB signaling [68].

### Impaired epithelial integrity leads to intestinal barrier dysfunction

Recent studies have shown that the disruption of intestinal barrier integrity is not only a consequence of IBD but may also be a key driving factor in the onset and progression of the disease. Multiple mechanisms are involved in this process, including increased epithelial cell apoptosis, abnormal expression of tight junction proteins, altered intestinal permeability, and endoplasmic reticulum stress.

Intestinal epithelial cells (IECs) form the front-line defense of the intestinal barrier, and the integrity of their structure and function is crucial for maintaining intestinal homeostasis and preventing the onset of IBD. The intestinal barrier is primarily maintained by tight junctions (TJs) between epithelial cells, which regulate intestinal permeability and prevent the invasion of pathogens and harmful substances into the submucosal layer. In patients with IBD, the abnormal expression or distribution of TJ proteins, such as claudin-1, zonula occludens-1 (ZO-1), and occludin, is a key factor contributing to increased intestinal permeability. Inflammatory stimuli such as LPS downregulate the expression of these proteins through the TLR4/NF-κB and myosin light chain kinase (MLCK)/myosin light chain (MLC) signaling pathways, while sulforaphane (SA) can restore TJ protein expression and barrier function by inhibiting both pathways [70]. Similarly, inulin alleviates LPS-induced epithelial barrier damage by upregulating the expression of claudin-1, claudin-2, and occludin [71]. It is worth noting that the expression of claudin-2 has a dual effect: under normal conditions, it participates in ion transport, but its overexpression in inflammatory states may lead to a “leaky” barrier [72]. Protein tyrosine phosphatase T-cell protein tyrosine phosphatase (TCPTP) inhibits the expression of claudin-2 by regulating matriptase, and loss-of-function mutations of TCPTP are associated with increased claudin-2 expression in IBD patients [72]. Moreover, overexpression of mucin MUC13 exacerbates TJ dysfunction through the janus kinase 1 (JAK1)/signal transducer and activator of transcription 3 (STAT3) and rho-associated coiled-coil containing protein kinase 2 (ROCK2)/mitogen-activated protein kinase (MAPK) signaling pathways, and IL-22 can activate these pathways [73]. It is noteworthy that the disruption of tight junctions is not only a result of inflammation but may also be an early event in the pathogenesis of IBD. In the IL-10-deficient mouse model, depletion of mucin and a reduction in Akkermansia bacteria occurred prior to the onset of inflammation, suggesting that barrier dysfunction may be a triggering factor for IBD [74].

Apart from TJ disruption, increased epithelial cell death directly leads to the loss of barrier function. In IBD, epithelial cell death includes not only apoptosis but also novel forms of cell death, such as necroptosis [10]. Mitochondrial dysfunction is a key factor driving epithelial cell death: Drp1-mediated excessive mitochondrial fission leads to ROS accumulation and apoptosis, while steroidogenic acute regulatory (StAR) protein-related lipid transfer (START) domain-containing protein 7 (STARD7) protects barrier function by maintaining mitochondrial membrane stability [75, 76]. Interestingly, aloe vera polysaccharides(AGP) mitigate anoikis(apoptosis induced by the loss of cell–matrix contact) by activating the nuclear factor erythroid 2-related factor 2 (Nrf2)-mitochondrial axis, illustrating a new mechanism by which natural compounds regulate epithelial survival [77]. Moreover, the absence of cortactin promotes epithelial apoptosis by increasing Ras homolog gene family, member A (RhoA)/ROCK1-dependent actin-myosin contraction, highlighting the importance of cytoskeletal dynamics in barrier maintenance [72].

Dysregulation of immune-epithelial interactions further exacerbates barrier damage. Pro-inflammatory cytokines, such as TNF-α and IFN-γ, can directly disrupt the epithelial barrier. TNF-α impairs barrier function by increasing epithelial cell shedding and downregulating occludin expression [78, 79], whereas IFN-γ increases permeability by inducing STAT1-dependent TJ remodeling [80]. In contrast, IL-22 exerts protective effects by promoting epithelial regeneration and the expression of transporters [81]. Lipocalin-2 (LCN2), derived from immune cells, is upregulated in inflammatory states, but its function depends on the cell source: epithelial-derived LCN2 protects the barrier, while immune-derived LCN2 may promote inflammation [82]. Furthermore, epithelial cell-specific deletion of Csk kinase results in reduced occludin expression and increased susceptibility to colitis, suggesting a critical role of epithelial signaling pathways in immune regulation [79].

Phosphorylation of MLC mediated by MLCK is an important mechanism regulating intestinal permeability. Activation of MLCK leads to cytoskeletal contraction, disrupting the integrity of TJs. Studies have shown that long-term use of proton pump inhibitors (PPIs) upregulates MLCK expression via a p38 MAPK-dependent pathway, exacerbating experimental colitis [83]. In contrast, adrenal medullin (AM) alleviates barrier damage in a TNBS-induced colitis model by inhibiting the MLCK-p-MLC pathway [84]. These findings reveal the potential of MLCK as a therapeutic target.

The endoplasmic reticulum(ER) is a crucial organelle for protein synthesis, folding, and modification. When subjected to various stress stimuli, ER homeostasis is disrupted, triggering the unfolded protein response (UPR), which, in turn, affects intestinal barrier integrity [85, 86]. Studies have shown that ER stress not only directly damages intestinal epithelial cell function but also exacerbates intestinal inflammation by regulating processes such as inflammation and apoptosis [87, 88].

Multiple studies have confirmed that ER stress directly leads to intestinal barrier dysfunction. In a chronic restraint stress (CRS) model, ER stress is activated through the PERK pathway, upregulating phosphorylated eukaryotic initiation factor 2α (P-eIF2α) and CCAAT/enhancer binding protein (C/EBP) homologous protein expression, leading to damage of the jejunal and colonic mucosa and disruption of barrier function [85]. Similarly, in intestinal epithelial cells from IBD patients, excessive ER stress induces increased trypsin-like activity, which activates protease-activated receptors 2 and 4, disrupting barrier function and increasing intestinal permeability [89]. These findings suggest that ER stress is a key driver of intestinal barrier damage.

Persistent ER stress can trigger multiple cell death pathways. Studies have found that ER stress promotes apoptosis, as evidenced by the upregulation of apoptosis markers such as Caspase-3 and Bcl-2-associated X protein (Bax)/B-cell lymphoma 2(Bcl-2) [85]. Additionally, ER stress activates the NOD-like receptor pyrin domain-containing 3 (NLRP3) inflammasome and the pyroptosis marker GSDMD, further exacerbating intestinal inflammation [85]. In goblet cells, elevated MUC2 mucin expression and misfolding lead to ER stress, triggering reactive oxygen species production and apoptosis, ultimately disrupting the integrity of the mucus barrier [90]. These mechanisms collectively lead to the loss of intestinal epithelial cells and compromised barrier function.

ER stress forms a vicious cycle with intestinal immune-inflammatory responses. Research has shown that ER stress-induced trypsin-like activity promotes the release of the inflammatory mediator C-X-C chemokine ligand 8 (CXCL8), thereby exacerbating the inflammatory process [89]. In the IBD model, the sphingosine-1-phosphate receptor 2 (S1PR2)/RhoA/ROCK1 pathway modulates ER stress, affecting vascular endothelial barrier function and classically activated macrophages (M1) macrophage polarization, further promoting intestinal inflammation [87]. These findings suggest that ER stress not only directly damages the epithelial barrier but also amplifies the inflammatory response by regulating immune cell function.

Through bioinformatics analysis and experimental validation, six key genes related to ER stress (epidermal growth factor receptor (EGFR), mesenchymal-epithelial transition factor (MET), insulin receptor (INSR), reticulophagy regulator 1 (RETREG1), MCL1, and basigin (BSG)) were identified, which are closely associated with pathways such as immune response and oxidative phosphorylation [86]. In the LPS-induced carcinoma of the colon-2 (Caco-2) cell model, the aberrant expression of these genes was associated with barrier dysfunction and ER stress activation, providing potential biomarkers for the diagnosis and treatment of IBD.

### Central role of gut microbiota in IBD

The normal gut microbiota is composed of various microorganisms, including bacteria, fungi, and viruses, which maintain gut homeostasis through metabolic products, immune regulation, and barrier function [91, 92]. However, dysbiosis is commonly observed in IBD patients, characterized by decreased microbial diversity, a reduction in beneficial bacteria, and an increase in potential pathogens [93–95]. This dysbiosis not only affects the bacterial composition but also involves changes in the fungal microbiota and influences disease progression through cross-species interactions between microorganisms [91, 92].

Multiple studies have confirmed significant dysbiosis of the gut microbiota in IBD patients. In the bacterial community, core microbiota such as Firmicutes and Bacteroidetes are reduced, while Proteobacteria, including Escherichia-Shigella and Enterococcus, are significantly enriched [93, 94, 96]. Although the fungal microbiota constitutes a smaller proportion, it plays a decisive role in regulating bacterial composition and mucosal immunity. For example, the interaction between Candida albicans and Salmonella may influence the pathology of IBD [91, 92]. This dysbiosis is not only associated with disease severity [97], but may also exacerbate intestinal inflammation by altering microbial functions, such as the enrichment of virulence factors [95].

Short-chain fatty acids (SCFAs) derived from the gut microbiota are not only the primary energy substrate for colonic epithelial cells but also serve as key anti-inflammatory messengers. A reduction in butyrate-producing bacteria, such as Faecalibacterium prausnitzii and Alistipes shahii, weakens the production of SCFAs, leading to the downregulation of HIF-1α signaling, loss of epithelial hypoxic zones, and ultimately the disruption of barrier integrity [97–99]. On the other hand, the microbiota influences the progression of IBD by regulating autophagy pathways and oxidative stress responses, with ROS scavengers, such as cerium oxide nanoparticles (CeO2@S100), showing therapeutic potential [100, 101].

The gut microbiota significantly influences the responses of T and B cells. In T cell receptor α subunit knockout (TCRα-/-) mice, Bacteroides vulgatus induces the production of Th2-type cytokines by colonic CD4 + T cell receptor ββ dimer (TCRββ) dimeric T cells, leading to colitis, whereas dietary modifications that alter the gut microenvironment can prevent disease progression [102]. Enterotoxigenic bacteroides fragilis (ETBF) induces a Th17 immune response through the activation of the Stat3 signaling pathway, with this activation persisting in immune and epithelial cells and relying on sustained ETBF colonization [103]. These findings emphasize the critical role of specific microbial species in driving aberrant immune responses.

Probiotics, such as Lactobacillus and Bifidobacterium, can regulate the cytokine profile, promoting the production of anti-inflammatory cytokines and inhibiting the expression of pro-inflammatory cytokines [66, 104–107]. Studies have shown that probiotic treatment can increase the levels of anti-inflammatory cytokines, such as IL-10, while reducing the levels of pro-inflammatory cytokines like TNF-α and IL-6, thereby improving gut barrier function [66, 105, 106]. SCFAs themselves can also inhibit histone deacetylases through the G protein-coupled receptor 109A (GPR43/109A) signaling pathway, further stabilizing the chromatin accessibility of the IL-10 promoter region [108].

Dysbiosis can serve as an initiating factor that weakens the barrier, or it can further disrupt the balance due to barrier damage. Antibiotic-induced dysbiosis leads to a decrease in the expression of ZO-1 and occludin, while the traditional Japanese medicine Saireito restores barrier function by modulating the gut microbiota composition [109]. Multistrain probiotics, including L. rhamnosus, enhance the barrier's resistance to LPS-induced damage by upregulating TJ protein expression and inhibiting NF-κB activation [110]. Notably, specific strains, such as Bifidobacterium pseudolongum, excessively proliferate and disrupt the barrier in Allergin-1-deficient mice, while neomycin can reverse this effect, indicating that immune receptors influence barrier function by modulating the microbiota composition [111]. Pathogenic bacteria, such as invasive Escherichia coli (AIEC), increase permeability and promote IBD progression by targeting TJ proteins, such as claudin-2 and occludin [112]. In contrast, probiotics such as Faecalibacterium prausnitzii and Lactobacillus rhamnosus CNCM I-3690 repair barrier function by enhancing mucin secretion and inhibiting inflammatory cytokines, such as IL-1β and IL-4 [113, 114].

### Role of oxidative stress and redox signaling in the pathogenesis of IBD

**Fig. 2:**
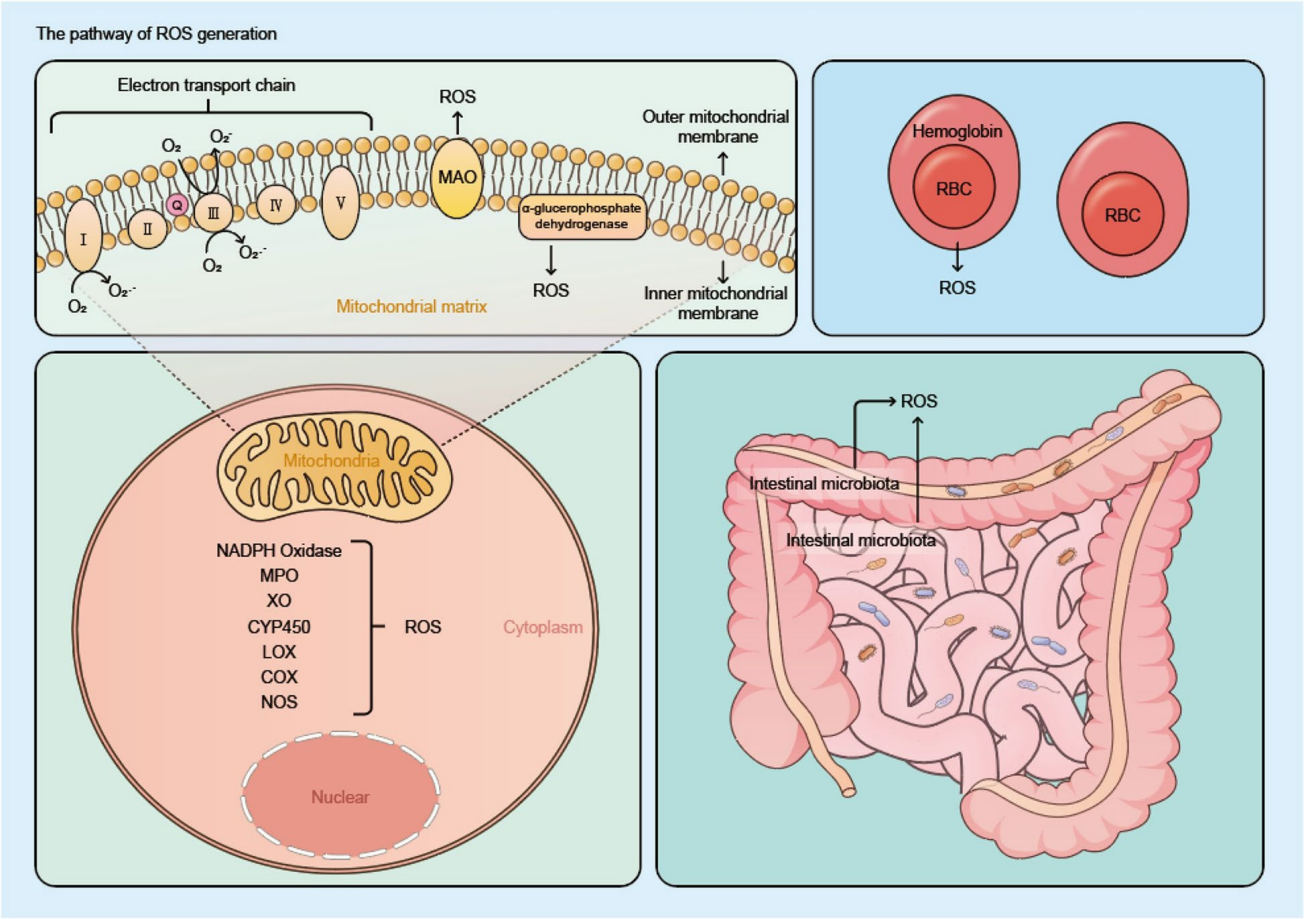
Schematic representation of the pathways of ROS production. The production of ROS occurs through various endogenous pathways, including the mitochondrial electron transport chain, MAO, succinate dehydrogenase, NADPH oxidase, MPO, XO, CYP450, LOX, COX, NOS, and hemoglobin within red blood cells, as well as through exogenous pathways such as intestinal microbiota. Note: ROS: Reactive oxygen species; O₂: Oxygen; O₂•.^−^: Superoxide anion; Complexes I–V: Mitochondrial Electron Transport Chain Complexes I–V; MAO: Monoamine oxidase; RBC: Red blood cell; MPO: Myeloperoxidase; XO: Xanthine oxidase; CYP450: Cytochrome P450; LOX: Lipoxygenase; COX: Cyclooxygenase; NOS: Nitric oxide synthase. (Figure was created by figdraw.com)

ROS refers to a group of highly reactive oxygen-derived species, including oxygen free radicals such as superoxide anion (O_2_•^−^), hydroxyl radicals (OH•), peroxy radicals (RO2•), and alkoxy radicals (RO•), as well as non-radicals such as hypochloric acid (HOCl), singlet oxygen (^1^O_2_), and hydrogen peroxide (H_2_O_2_). To ensure the efficient progression of redox reactions, cells compartmentalize these reactions into distinct subcellular compartments. The substrates and oxidases utilized by these compartments vary, leading to differences in reaction rates and intensities. As a result, the concentration and types of ROS generated as byproducts of redox reactions differ between compartments. For example, quantitative studies on thiol oxidation have shown that the thiol/disulfide ratio varies across different subcellular compartments [115] (Fig. 2).

Under physiological conditions, ROS play a critical role as important signaling molecules in maintaining intestinal homeostasis. As key mediators of the interaction between intestinal epithelial cells and the microbiota, ROS maintain intestinal health through multiple mechanisms, including the regulation of immune defense, barrier function, stem cell dynamics, and microbiota composition [116–118]. Several studies have revealed the central role of the nicotinamide adenine dinucleotide phosphate (NADPH) oxidase family and mitochondrial-derived ROS in antimicrobial defense, cellular signal transduction, and metabolic regulation [34, 117, 119]. Moderate levels of ROS promote the proliferation of intestinal stem cells. In the Drosophila model, appropriate levels of ROS activate the metabolic and differentiation programs of intestinal stem cells, promoting tissue repair and intestinal regeneration [120]. Studies have shown that physiological levels of ROS can activate the Notch signaling pathway, promoting a balance in the differentiation of intestinal stem cells into absorptive and secretory cells [121]. During the differentiation of esophageal and intestinal epithelial cells, ROS and the bone morphogenetic protein (BMP) signaling pathway form a positive feedback loop [122]. Activation of the BMP signaling pathway induces ROS production, which in turn further enhances the signaling transduction of the BMP pathway, collectively promoting the transition of basal progenitor cells into differentiated cells [122]. Especially during the differentiation of esophageal squamous epithelium, BMP-driven ROS production activates Nrf2-mediated oxidative stress response, a mechanism crucial for normal epithelial differentiation [122]. Furthermore, moderate levels of ROS serve as key mediators of innate immune defense. Studies have shown that peroxynitrite generated by the coordinated action of inducible nitric oxide synthase (iNOS) and NOX1 effectively suppresses excessive proliferation of ileal bacteria, preventing the reflux of colonic microbiota [116].

In general, due to the presence of the oxidative and antioxidant systems in cells, ROS production and elimination are maintained at stable levels. However, under stress conditions, the accumulation of ROS can cause damage to DNA, proteins, and lipids, which in turn affects the cell cycle, leading to cell death and the onset of inflammation. Changes in ROS levels are a critical early event in the development of IBD. Increased ROS production has been observed in the intestinal tissues of IBD patients and mouse models of colitis, which subsequently leads to damage to the intestinal mucosa. First, pro-inflammatory cytokines can activate NOX and iNOS, leading to the production of ROS by IECs, neutrophils, and macrophages. At the same time, excessive ROS accumulation activates inflammatory/immune responses through signaling pathways such as NF-κB, MAPK, and STAT3, resulting in increased expression and secretion of pro-inflammatory cytokines. ROS regulates the maintenance of the mucus layer and bidirectional communication with the symbiotic microbiota, thus promoting the integrity of the epithelial barrier. ROS also disrupts cytoskeletal proteins, alters tight junctions in the intestinal epithelium, and increases intestinal permeability, leading to the infiltration of toxins and other harmful substances into the intestinal wall, thus exacerbating intestinal inflammation.

In IBD, ROS affects the differentiation of intestinal immune cells. Changes in ROS levels can influence the differentiation of T cells, leading to an increased differentiation of T cells towards Th1 and Th17 subtypes in IBD intestinal tissues. Mitochondrial ROS (MtROS) acts as a third signal, directly enhancing the T-box transcription factor 21 (T-bet)/STAT1 pathway, increasing IFN-γ secretion, and driving Th1 responses [123]. NOX2-ROS, on the other hand, suppresses STAT5 phosphorylation and GATA-3 expression, relieving the Th2 bias against Th1 and indirectly promoting Th1 differentiation. Conversely, the clearance of mtROS using MitoQ or the genetic deletion of NOX2 results in reduced IFN-γ production and inhibited Th1 differentiation, suggesting that ROS from different sources positively promotes Th1 differentiation in IBD. In contrast, there is broad consensus regarding the indirect effect of ROS on Th17 cell differentiation. Studies have shown that calcium-dependent mitochondrial ROS are essential for antigen-driven T cell activation [124]. MtROS can promote downstream T cell receptor (TCR) signaling, which is a prerequisite for Th17 differentiation. HIF-1 is a key inducer of the metabolic shift dominated by glycolysis. It enhances TH17 differentiation by directly activating RORγt transcription and promoting the binding of RORγt to p300. It also reduces the protein levels of the Treg regulatory factor forkhead box P3 (Foxp3), inhibiting the development of Treg cells. HIF-1 not only drives the generation of TH17 cells but also ensures their sustained presence [125]. ROS indirectly promotes Th17 differentiation through HIF-1. Studies have shown that ROS primarily stabilizes HIF-1α by inhibiting prolyl hydroxylase (PHD) activity [126, 127]. Moreover, in various cell types, ROS can enhance HIF1A transcription by activating NF-κB [128, 129]. In the intestine, an increase in ROS was observed in Caco-2 intestinal epithelial cells treated with H_2_O_2_, accompanied by elevated HIF-1α and RelA/RelB levels [130]. However, no studies have yet demonstrated that intestinal ROS can enhance HIF1A transcription through the activation of NF-κB.

ROS influences the polarization of intestinal immune cells in IBD patients. In IBD, ROS has been shown to promote the polarization of intestinal macrophages toward the M1 phenotype [131], though the precise mechanism remains unclear. In both IBD patients and animal models, an increase in the number of M1 macrophages and a significant elevation in serum levels of pro-inflammatory cytokines, including TNF-α, IL-1β, and IL-6, have been observed, with ROS playing a pivotal role as a signaling molecule in triggering and amplifying the inflammatory response. This causal relationship is particularly critical in the pathogenesis of IBD, as excessive polarization and activation of M1 macrophages are key contributors to sustained intestinal inflammation and tissue damage. In a study involving HIF1 transgenic mice, DSS treatment led to an increase in the recruitment of M1 macrophages in wild-type mice, while mtROS production was elevated in HIF1 transgenic mice, inducing a shift of macrophages toward M2 polarization in the intestines [132]. The selective elimination of mtROS reduced the induction of M2 macrophages in HIF1 transgenic mice, and further investigation suggested that mtROS-induced M2 macrophage polarization is mediated through NF-κB activation. Although these results appear to contradict previous studies reporting ROS-induced M1 macrophage polarization, they actually reveal the dual role of mtROS under varying dosages and immune environments. In this study, the production of mtROS is derived from low-level, sustained ROS signaling generated by reverse electron transfer (RET) following partial inhibition of ATP synthase, whereas typical IBD pathology is characterized by high levels of ROS. Studies have shown that the amount and intensity of ROS production play a decisive role in the balance between M1 and M2 macrophage polarization. Low levels of ROS promote the expression of M2-related genes, while excessive ROS drive the high expression of M1 markers [133, 134]. Furthermore, upon NF-κB activation, IL-10, IL-4, and IL-6 (mildly) are upregulated, while the expression of pro-inflammatory cytokines such as TNF-α and IL-1β does not show a significant increase. This may be related to the differential activation of NF-κB target genes. Previous studies have indicated that NF-κB activates different target genes under various stimuli: in M1 polarization, the p65/c-Rel subunit dominates the expression of pro-inflammatory genes, while in M2 polarization, the RelB or p50/p50 subunits govern the activation of anti-inflammatory or suppressive programs [135].

ROS can modulate the interactions between immune cells and IECs in the colon during IBD. Under physiological conditions, ROS contribute to maintaining intestinal homeostasis by providing antimicrobial defense and supporting epithelial barrier function [136]. However, pathological accumulation of ROS leads to mitochondrial oxidative damage [137] and apoptosis of IECs [138]. IECs contribute to the formation of the chemical barrier through the secretion of ROS and other substances [139]. However, excessive ROS during chronic inflammation exacerbate immune responses by activating the NF-κB pathway [140] and promoting immune cell infiltration. Tregs and Th1 cells modulate IECs by secreting IL-10 and IFN-γ. IL-10 helps maintain IEC homeostasis [141], whereas IFN-γ synergizes with TNF-α to activate the JAK/STAT pathway [142], further impairing IEC function. In recent years, ferroptosis, a form of regulated cell death dependent on iron and lipid peroxidation, has been demonstrated to play a key role in intestinal oxidative stress during IBD. This process is initiated by radical chain reactions of polyunsaturated phosphatidylethanolamines (PUFA-PE), accompanied by a sustained burst of ROS [143]. Specifically, ferritinophagy releases Fe^2^⁺, which participates in Fenton-like reactions to generate OH•, directly amplifying lipid peroxidation [144]. Meanwhile, Acyl-coA synthetase long-chain family member 4 (ACSL4) esterifies PUFAs into phospholipids, expanding the pool of oxidizable substrates, while the 15-lipoxygenase(15-LOX)/phosphatidylethanolamine-binding protein 1 (PEBP1) complex catalyzes the formation of specific peroxidized PE species, serving as a persistent source of ROS [145]. Recent studies have further revealed that ACSL4 is highly expressed in the fibroblasts of the IBD gut, actively secreting phospholipids rich in PUFAs, which, upon uptake by epithelial cells, lower the threshold for ferroptosis, thus forming a cross-cellular ROS network between fibroblasts, epithelial cells, myeloid cells, and endothelial cells [146]. Within this network, NOX2-MPO(myeloid) and XO/eNOS (endothelial) amplify ROS signals in a secondary manner, driving the ferroptosis cascade. Moreover, the imbalance of the antioxidant system further exacerbates lipid ROS accumulation: when the glutathione peroxidase 4 (GPX4), ferroptosis suppressor protein 1 (FSP1)-coenzyme Q10 (CoQ10), and dihydroorotate dehydrogenase (DHODH)-CoQ10 axes are restricted, lipid peroxides cannot be reduced, leading to the continuous accumulation of ROS [147]. In human and mouse IBD samples, ferroptosis-related genes are significantly upregulated, total ROS and lipid ROS levels are elevated in parallel, and the final product 4-hydroxynonenal(4-HNE) accumulates in large amounts [148]. Intervention with Ferrostatin-1, Liproxstatin-1, or iron chelators resulted in a simultaneous decrease in lipid peroxidation signaling, malondialdehyde (MDA) levels, and tissue inflammation scores, confirming that the “ferroptosis-lipid peroxidation-ROS” axis is one of the primary sources of oxidative stress in IBD, rather than a secondary phenomenon [149]. To address the ROS burst driven by ferroptosis and the imbalance of iron homeostasis, a study has developed a macrophage-targeting polysaccharide iron nanoenzyme, which possesses dual enzyme activities resembling catalase and peroxidase, capable of scavenging ROS, inhibiting lipid peroxidation, and controllably releasing Fe^2^⁺ to correct systemic iron deficiency [150].

The impact of ROS on gut barrier function primarily involves multiple aspects. Existing studies suggest that ROS from different sources have distinct effects on cells. ROS generated by NADPH oxidase affect cell proliferation and differentiation by modulating cell signaling, while excessive suppression of NADPH oxidase may impair its role in maintaining gut barrier stability. In contrast, excessive ROS in mitochondria induce apoptosis by increasing Bax expression [151]. In intestinal inflammation, the accumulation of ROS leads to the depletion of antioxidant enzymes, thereby directly disrupting the gut barrier [152]. ROS can also indirectly disrupt the gut barrier by significantly reducing the expression of tight junction proteins such as ZO-1, Occludin, and Claudin-4 [153]. In contrast, activation of the Nrf2/Kelch-like ECH-associated protein 1 (Keap1) antioxidant pathway upregulates the expression of tight junction proteins, thereby repairing barrier function [154]. Moreover, ROS can impair the epithelial barrier by disrupting the mucus layer, a protective barrier on the intestinal epithelial surface composed of mucins such as MUC2 secreted by IECs. This layer not only restricts bacterial contact with the intestinal epithelium but also provides nutrients and adhesion sites for the microbiota. ROS disrupt the integrity of the mucus layer by downregulating MUC2 expression [155], weakening its physical barrier function against intestinal microorganisms. The interplay between gut microbiota dysbiosis and ROS also affects the gut barrier: On one hand, dysbiosis increases gut ROS levels, inducing stress and barrier damage [156]; on the other hand, excessive ROS disrupt the balance of the gut microbiota, subsequently impairing the gut barrier.

ROS can promote intestinal fibrosis. Under inflammatory conditions, ROS enhance the fibrotic response by promoting the generation of extracellular matrix (ECM) and inhibiting its degradation [157]. The abnormal deposition of ECM forms the basis for intestinal wall thickening and stenosis, with transforming growth factor-β (TGF-β)/Smad signaling playing a significant role. TGF-β1 promotes ROS generation and activates related fibrotic signaling pathways by inducing the expression and activation of NOX4 [157, 158]. The accumulation of ROS triggers redox imbalance, further enhancing the pro-fibrotic effect of TGF-β1 [159], thus creating a vicious cycle. Furthermore, ROS regulate the activity of multiple transcription factors, such as Nrf2, NF-κB, p53, HIF, and Smad proteins, by activating protein kinases, inhibiting phosphatases, or directly interacting with transcription factors. These factors play crucial roles in fibrosis and cancer development [157]. ROS also promote fibrosis by inducing epigenetic changes such as DNA methylation, with the associated DNA hypermethylation downregulating several anti-fibrotic genes [157], thereby exacerbating the progression of intestinal fibrosis.

## Genetic and environmental factors in IBD

A comprehensive investigation of modifiable risk factors underlying IBD is considered fundamental to enhancing disease prevention strategies and therapeutic outcomes. At the genetic level, genome-wide association studies (GWAS) have identified over 240 IBD susceptibility loci implicated in critical pathways including immune regulation, intestinal barrier function, and autophagy [13, 14]. Both monogenic defects [160, 161] and polygenic cumulative effects [162, 163] have been demonstrated to constitute the genetic basis of disease pathogenesis, with this susceptibility being influenced by ethnicity-specific [164] and age-specific [165, 166] variations as well as epigenetic regulatory mechanisms [167, 168]; At the environmental and lifestyle level, dietary patterns [169], environmental pollutants [170, 171], physical activity habits, and smoking/alcohol consumption [172–174] have been shown to serve as key external triggers that precipitate or exacerbate disease through modulation of intestinal microbiota homeostasis, disruption of epithelial barrier function, and activation of oxidative stress and inflammatory pathways. More importantly, genetic and environmental factors have been found to interact synergistically through epigenetic modifications and microbiota-immune crosstalk, thereby jointly influencing disease susceptibility, phenotypic heterogeneity, and therapeutic responsiveness. Herein, the genetic susceptibility mechanisms of IBD, the specific impacts of environmental and lifestyle factors, and their interactive patterns are systematically reviewed, with particular emphasis on the mechanistic pathways of modifiable factors, with the aim of providing practical guidance for the development of personalized prevention strategies and therapeutic interventions.

### Genetic susceptibility to IBD

The occurrence of IBD is closely associated with genetic factors, and studies have shown that specific genetic variants may lead to immune system dysfunction, thereby triggering intestinal inflammation. Extensive GWAS have identified over 240 risk loci associated with IBD, with these genetic variants distributed across several critical pathways, including immune regulation, intestinal barrier function, autophagy, and cell signaling [13, 14]. Nucleotide-binding oligomerization domain containing 2 (NOD2) is the first confirmed susceptibility gene for CD, and its mutation is significantly associated with the risk of CD onset [175, 176]. The R381Q variant of the IL23R gene provides a protective effect for CD, and functional studies have shown that this variant reduces IL-23 receptor function, thereby weakening the Th17 cell-mediated inflammatory response [177]. Variants in autophagy-related genes such as autophagy-related gene 16-like 1 (ATG16L1) and immunity-related GTPase M (IRGM) also affect IBD susceptibility, particularly in CD [178, 179]. Moreover, transcriptome-wide association studies (TWAS) combine gene expression profiles with GWAS variants to further identify IBD candidate genes: On one hand, risk variants in intestinal macrophages drive gene expression differences associated with inflammation and immune cell differentiation [180, 181], on the other hand, effect genes involved in stress-immune regulation were identified in the hypothalamus and colonic epithelium, suggesting the role of the neuro-immune axis [182]. The macro–micro genetic map constructed thus provides a theoretical foundation for precise IBD diagnosis and treatment, with future multi-omics and multi-population studies expected to expand this framework.

The genetic susceptibility of IBD has been shown to be closely related to the expression of specific genes in various tissues and cell types, revealing the crucial role of gene function in disease pathogenesis. The secretion of antimicrobial peptides by Paneth cells is regulated by susceptibility genes and depends on microbiota interactions [183], while the loss of transcription factor EB (TFEB) in colonic epithelium weakens the antioxidant barrier [184]; leucine-rich repeat kinase 2 (LRRK2) [185, 186], autophagy genes ATG16L1/IRGM/NOD2 [187], G protein-coupled receptor 35 (GPR35)-extracellular signal-regulated kinase (ERK) [188] and CR6 interacting factor 1 (CRIF1)-mitochondria-TH17 [189] are associated with the neuro-immune, autophagy, inflammation, and metabolism axes, respectively. DNA methylation and microRNA-374a-5p (miR-374a-5p) epigenetically regulate macrophage function [190, 191]. In conclusion, the functions of specific genes in IBD involve intestinal epithelial cells and immune cells, with tissue- and cell-specific regulation of gene expression being key to the genetic susceptibility of IBD.

The genetic susceptibility of IBD presents a continuum, ranging from fully penetrant Mendelian inheritance (monogenic IBD) to the complex genetic forms involving the cumulative effects of multiple low-impact risk genes [192]. Monogenic IBD typically manifests as very early onset(very early onset inflammatory bowel disease (VEO-IBD)), caused by rare pathogenic variants in genes such as IL10R and X-linked inhibitor of apoptosis protein (XIAP) [193]. In contrast, common adult-onset IBD is primarily polygenic, involving the cumulative effects of hundreds of low-impact risk loci [166, 194]. Studies have found that there is partial overlap in the genetic backgrounds of monogenic and polygenic IBD, suggesting shared pathogenic pathways [195].

Epigenetic mechanisms, such as DNA methylation, mediate the regulation of gene expression by environmental factors. It has been found that widespread DNA methylation abnormalities exist in the intestinal mucosal cells of IBD patients, some of which are associated with IBD susceptibility loci [196, 197]. For example, the rs36221701 locus near the Smad3 gene exhibits allele-specific methylation and affects the expression levels of Smad3 [196]. These epigenetic alterations may serve as a molecular bridge between genetic susceptibility and environmental exposure, contributing to the onset and progression of IBD [198].

Variations in IBD susceptibility genes are closely associated with the phenotypic heterogeneity of the disease. For example, variations in NOD2 are associated with the fibrotic-stricturing phenotype of CD [175], whereas variations in the human leukocyte antigen (HLA) region affect the disease extent and severity of UC [199]. Genetic factors also influence treatment response, with polymorphisms in genes such as IL23R and TNFRSF1A being associated with responses to anti-TNF therapy [200]. These findings provide a molecular basis for personalized treatment of IBD.

Comparative studies across ethnic groups reveal significant differences in the genetic structure of IBD between European and Asian populations. While the genetic background of UC is relatively similar between the two populations, the susceptibility gene profile of CD differs significantly [178, 201]. For example, the association between NOD2 and autophagy pathway genes is weaker in East Asian populations, whereas the association between IL23R/Th17 pathway genes is more pronounced in East Asian populations [178, 202]. These differences may reflect variations in the response to environmental factors and evolutionary selective pressures among different populations.

Cross-age comparisons show that the genetic structures of pediatric and elderly IBD exhibit a “bipolar differentiation” VEO-IBD has the heaviest genetic burden: more than one-third of those with onset before age 6 carry single-gene defects, IL-10R mutations can be cured through hematopoietic stem cell transplantation [203–205], NOD2 biallelic mutations often lead to stricturing CD and high surgery rates [206],and rare defects such as lipopolysaccharide-responsive beige-like anchor protein (LRBA) and syntaxin-binding protein 2 (STXBP2) are also frequently observed [206, 207]; The risk further increases when associated with immunodeficiency syndromes such as Turner/Down syndrome [208]; Genetic variations in pediatric IBD patients are characterized by high molecular heterogeneity and random distribution [179]. In contrast, the genetic contribution to IBD in elderly patients is significantly reduced, with immunosenescence, dysbiosis, and comorbidities becoming predominant. The clinical phenotype is milder, but the incidence of infectious complications is high, requiring more age-specific risk models [209, 210]. Future efforts should integrate genomic, immune, and microbiological multidimensional data to construct a personalized diagnostic and therapeutic system that covers the entire lifespan.

### Impact of environment and lifestyle

The pathogenesis of IBD is complex, with environmental factors and lifestyle playing a significant role in the onset and progression of the disease. Numerous studies have shown that various environmental factors, such as dietary patterns, lifestyle habits, and environmental pollution, significantly affect the risk of IBD onset and its clinical manifestations.

**Fig. 3:**
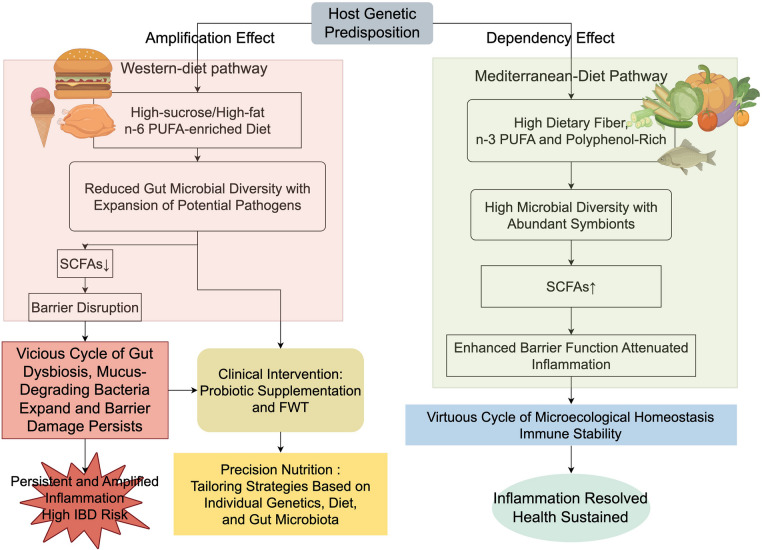
Host genetic predisposition modulates the diet–microbiome axis through “amplification” and “dependency” effects, critically determining IBD susceptibility and clinical outcome. Left track: a Western-style diet(high sucrose, saturated fat, n-6 PUFA) reduces microbial diversity and SCFA availability, expands pathobionts and mucus-degrading species, perpetuates barrier leakage, and locks genetically prone individuals into a vicious cycle of amplified inflammation. Right track: a Mediterranean-style diet(rich in dietary fiber, n-3 PUFA and polyphenols) increases symbiont abundance and SCFA production, reinforces barrier integrity, and induces immune homeostasis, establishing a virtuous micro-ecological cycle. Precision-nutrition interventions that integrate individual genetics, diet composition, and targeted probiotics can shift the balance toward dependency on protective microbiota, leading to inflammation resolution and sustained health even in high-risk genotypes. Note: PUFA: Polyunsaturated fatty acid; SCFAs: Short-chain fatty acids; FWT: Fecal microbiota transplantation; IBD: Inflammatory bowel disease. (Figure was created by figdraw.com)

Dietary habits, as a key environmental factor in the pathogenesis and management of IBD, have garnered widespread attention in recent years. Different dietary patterns significantly impact the gut microbiota, thereby modulating intestinal immune responses and influencing the onset and progression of IBD. Current research primarily focuses on two typical dietary patterns: the Mediterranean Diet (MD) and the Western diet, as well as the association between high-fat, high-sugar diets and IBD risk. First, the Mediterranean Diet, which emphasizes vegetables, fruits, whole grains, nuts, olive oil, and moderate fish intake, has been shown to enhance microbiota diversity, increase SCFA-producing bacteria, and maintain barrier function. Systematic reviews have demonstrated that high adherence to this diet is significantly associated with a reduced risk of IBD [211–213], and it also helps to supplement energy and micronutrients in pediatric patients [214]; Next, the Western diet, characterized by high consumption of red meat, processed foods, and high-fat, high-sugar content with insufficient fiber, promotes dysbiosis and an increase in pro-inflammatory bacteria. Epidemiological studies have confirmed its association with the onset of IBD and the exacerbation of active CD [15, 16]; Furthermore, high-fat, high-sugar diets contribute to the onset of IBD by enhancing oxidative stress [215], High sugar intake also exacerbates inflammation by lowering FGF-19 levels [216], Additionally, patients often experience insufficient fiber and vitamin intake due to disease symptoms and dietary restrictions [214, 217]; Furthermore, more than 50% of patients adjust their diet based on personal experience after diagnosis, often without professional guidance, which can lead to malnutrition and a decline in quality of life [218–220]; In summary, advocating for a high-fiber, anti-inflammatory Mediterranean diet, along with providing scientific dietary guidance, is a crucial component of comprehensive IBD management (Fig. 3).

**Fig. 4:**
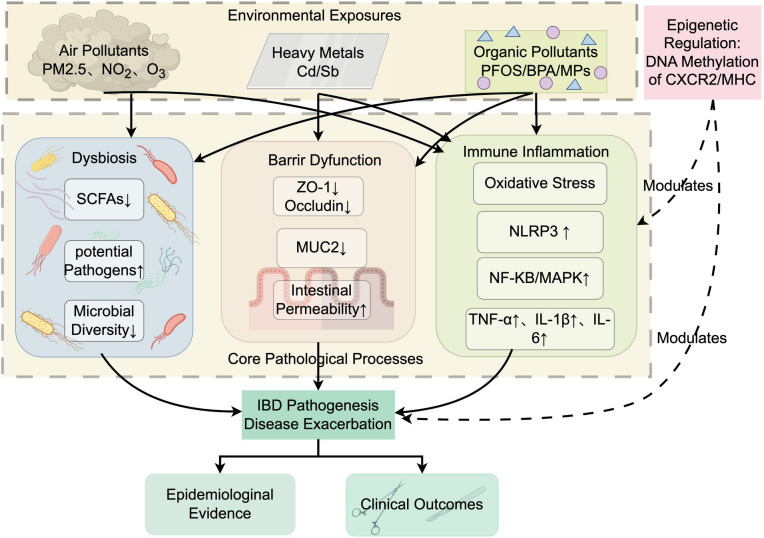
Environmental exposures (PM₂.₅, NO₂, Cd, PFOS, etc.) epigenetically down-regulate CXCR2 and MHC via DNA methylation, triggering dysbiosis, reduced barrier proteins (ZO-1, Occludin, MUC2), and oxidative stress. consequent NLRP3–NF-κB/MAPK activation elevates TNF-α, IL-1β and IL-6, increases intestinal permeability, and drives the core pathological cascade of IBD while worsening clinical outcomes; epidemiological evidence shows a positive dose–response relationship between exposure levels and disease exacerbation risk. Note: PM2.5: Fine particulate matter(aerodynamic diameter ≤ 2.5 μm); NO₂: Nitrogen dioxide; O₃: Ozone; Cd: Cadmium; Sb: Antimony; PFOS: Perfluorooctanesulfonic acid; BPA: Bisphenol A; MPs: Microplastics; SCFAs: Short-chain fatty acids; ZO-1: Zonula occludens-1; MUC2: Mucin 2; NLRP3: NOD-like receptor family pyrin domain-containing 3; NF-κB: Nuclear factor κ-light-chain-enhancer of activated B cells; MAPK: Mitogen-activated protein kinase; TNF-α: Tumor necrosis factor-α; IL: Interleukin; IBD: Inflammatory bowel disease; CXCR2: C-X-C motif chemokine receptor 2; MHC: Major histocompatibility complex. (Figure was created by figdraw.com)

Environmental pollution, as a widespread phenomenon in modern society, has a broad and profound impact on human health, particularly playing a significant potential role in the onset and development of IBD (Fig. 4). In recent years, epidemiological and translational medicine research has continually revealed how air pollution, heavy metals, and various chemicals affect gut health through multiple mechanisms, promoting the onset and exacerbation of IBD. Firstly, long-term exposure to air pollutants (PM2.5, NO₂, ozone) increases intestinal permeability, systemic inflammation, and dysbiosis [221], The UK Biobank cohort has confirmed that PM2.5 and NO₂ significantly increase the risk of UC [222], Other studies have also revealed a correlation between PM2.5 exposure and increased rates of intestinal resection and mortality [223]; Secondly, the heavy metal cadmium reduces mucus secretion, causes loss of goblet cells, and excessively activates the Notch pathway [224], Antimony also induces barrier damage and dysbiosis [225]; Thirdly, synthetic chemicals such as perfluorooctane sulfonate (PFOS), bisphenol A(BPA), phthalates, and microplastics also have an impact on IBD. PFOS exposure disrupts intestinal barrier function and interferes with key proteins and signaling pathways [226], BPA and its alternatives disrupt glucose-lipid metabolism and induce inflammatory responses [227]; Plastic pollutants, particularly microplastics and nanoplastics, induce intestinal barrier dysfunction, dysbiosis, and metabolic abnormalities [170, 228, 229]. At the mechanistic level, environmental pollutants first increase intestinal permeability, allowing bacteria and their products to cross the intestinal barrier and activate the immune system, triggering chronic inflammation [230, 231]; Secondly, oxidative stress induced by pollutants activates signaling pathways such as NLRP3 inflammasome, MAPK, and NF-κB, promoting the production of pro-inflammatory cytokines and exacerbating intestinal damage [232, 233]; Furthermore, pollutants reduce beneficial bacteria such as those responsible for short-chain fatty acid production, promoting the overgrowth of harmful bacteria and disrupting microbial homeostasis [234]; Additionally, pollutants can alter the DNA methylation of regions such as C-X-C chemokine receptor type 2 (CXCR2) and major histocompatibility complex (MHC), epigenetically regulating immune and inflammatory levels [235]. Notably, air pollution and pesticide exposure resulting from urbanization are significantly associated with spatial clustering of childhood IBD incidence [236–238], Furthermore, the effects on barrier function and immune regulation explain the rising incidence of IBD in industrialized regions [239]. In conclusion, environmental pollution disrupts microbial ecology and immune homeostasis through multiple pathways, providing modifiable risk targets for the prevention, prediction, and intervention of IBD.

Gene-environment interactions play a critical role throughout the pathogenesis of IBD. Mendelian randomization has confirmed a significant association between PM2.5 exposure and increased genetic susceptibility to UC [240], Immigrant studies also indicate that individuals migrating from low-incidence to high-incidence areas have an elevated risk of IBD [241, 242], while high birth order (≥ 3), due to early exposure to infections, is associated with a reduced risk of IBD [243], These macro-epidemiological findings underscore the importance of the synergistic role of environmental changes and genetic background. At the mechanistic level, aluminum intake induces differential inflammatory responses in patients with different genetic backgrounds, suggesting that gene-environment interactions determine the gut's detoxification and inflammatory amplification capacity [244]; In animal models, NOD2 and Atg16l1 mutant mice exhibit specific immune cell composition and cytokine profile changes in a natural microbiome environment, directly demonstrating that both genetics and the environment jointly regulate gut immunity [245]; Clinical combined risk scores further highlight that environmental exposures during early life have a greater impact on the age of IBD onset than genetic factors, emphasizing that the environment amplifies genetic risk through epigenetics, microbiota, and barrier signaling pathways, playing a dominant role in disease initiation [246].

Complex interactions exist between dietary factors, the gut microbiome, and genetic susceptibility. Western diets high in sugar, fat, and n-6 PUFA disrupt the gut barrier by increasing pro-inflammatory mediators, whereas Mediterranean diets rich in dietary fiber, plant polyphenols, and n-3 PUFA enhance microbiota diversity and promote the production of anti-inflammatory metabolites, such as SCFAs, thereby alleviating intestinal inflammation [247–249]; Children with IBD generally have insufficient fiber intake, and supplementation with fiber and probiotics can restore symbiotic bacteria abundance, enhance barrier function, and reduce inflammation [250, 251], Moreover, IL-10 knockout mice fed a low-fiber diet show mucosal layer degeneration and severe colitis, directly confirming that genetic background amplifies the dietary effects [252]. Furthermore, a reduction in symbiotic bacteria such as Faecalibacterium prausnitzii and overgrowth of pathogenic bacteria have been observed in the gut of IBD patients, with host-associated gene mutations impairing the recognition and clearance of the gut microbiome. The interactions among bacteria, fungi, and viruses disrupt immune homeostasis, leading to persistent inflammation [253–258]; Moreover, fiber deficiency promotes the proliferation of mucus-degrading bacteria, exacerbating barrier damage and amplifying inflammation [252]. Clinical and animal studies have demonstrated that microbiota reconstruction through probiotics, prebiotics, exclusion diets, or fecal microbiota transplantation can effectively alleviate inflammation. However, the efficacy of these interventions varies due to individual genetic backgrounds, dietary habits, and baseline microbiome differences [251, 259–262]. This suggests the need for the development of precision nutrition-microbiome intervention strategies. In summary, diet, by shaping the gut microbiome and its metabolic functions, along with host genetic susceptibility, jointly determine the risk and progression of IBD. In the future, integrating genetic information and developing personalized nutrition and microbiome therapeutic strategies will be essential to achieve the goal of precision medicine for IBD [251, 252, 261].

The role of exercise and physical activity in IBD management has garnered increasing attention in recent years. Exercise not only serves as a non-pharmacological treatment but also exerts potential benefits by modulating the immune system. Repeated moderate-intensity exercise can induce functional changes in immune cell responsiveness to bacterial stimuli [263], and moderate exercise can improve gastrointestinal symptoms and alleviate stress [264]; Structured exercise programs significantly enhance cardiovascular function, bone density, muscle mass, and sleep quality [265–267], Aerobic, resistance, and whole-body vibration training can alleviate fatigue, anxiety, and depression, improving quality of life [265, 268, 269]. Furthermore, females [270], and patients in active disease phases [271] typically engage in insufficient physical activity, with pain [272, 273], fatigue [272, 273] and psychological disorders [273] being the primary barriers, requiring individualized programs and professional education. Furthermore, low to moderate-intensity exercise is safe and effective. High-intensity exercise requires careful monitoring for gastrointestinal discomfort and enhanced nutritional support [274], Currently, a unified prescription guideline is lacking, and in the future, the optimal intensity, frequency, and modality should be clearly defined [275, 276]. In summary, exercise provides a safe and feasible adjunct to IBD management through multiple immune-metabolic-psychological pathways. However, large-scale long-term follow-up studies are needed to refine the evidence and guidelines [277].

Smoking and alcohol consumption, as environmental and lifestyle factors, have differential effects on IBD, presenting both positive and negative impacts. The effects of smoking are well-established and contradictory—significantly increasing the risk of CD, disease activity, and the need for surgery/hospitalization [173, 278], However, smoking demonstrates a “protective” effect in UC [173, 279], and the risk of relapse in UC increases immediately after smoking cessation [280, 281], It may also amplify the effects of certain genetic variations through gene-environment interactions [282]; Evidence on alcohol consumption is still insufficient, but genetic predisposition analysis suggests a link between alcohol consumption and IBD risk [283, 284], The mechanisms involve microbiome disruption, barrier dysfunction, and immune activation [285], Clinical observations indicate that high alcohol consumption exacerbates symptoms and increases the risk of relapse, with varying effects from different types of alcohol (red wine may be anti-inflammatory, while white wine/beer may be pro-inflammatory) [284], It also interferes with drug metabolism and increases side effects [285]. Therefore, lifestyle interventions should prioritize smoking cessation as the primary measure for CD and carefully manage the timing of smoking cessation in UC, while limiting total alcohol intake and selecting the type of alcohol to optimize IBD treatment response and long-term prognosis.

## Emerging therapeutic prospects and strategies

The therapeutic landscape of IBD has evolved from conventional non-specific anti-inflammatory approaches to an era of precision-targeted therapy, with the current therapeutic framework being centered on biologics and small molecule inhibitors, which has led to significant improvements in clinical outcomes for patients with moderate-to-severe disease. Regarding optimization of existing therapeutics, anti-TNF-α agents [286], integrin inhibitors [287], and IL-12/23 inhibitors [288] have been established as first- or second-line treatment options for moderate-to-severe IBD through their targeting of critical pathways such as inflammatory cascades and immune cell trafficking, while the implementation of therapeutic drug monitoring (TDM), personalized dosing regimens, and novel drug delivery systems has been shown to further enhance therapeutic stability and safety profiles; JAK inhibitors [289] and other small molecule therapeutics have emerged as important alternatives for patients who have experienced biologic therapy failure or intolerance, offering advantages of oral administration convenience and multi-target anti-inflammatory effects. Nevertheless, a subset of patients continues to face therapeutic challenges associated with primary/secondary treatment failure, adverse reactions, and disease heterogeneity, which has driven the development of emerging therapies based on pathological mechanisms. Emerging therapeutic strategies, ranging from immune modulation and intestinal barrier restoration to microbiota intervention, metabolic pathway targeting, and ROS scavenging, have been developed to address core pathogenic mechanisms of IBD, thereby establishing a multi-dimensional, precision-oriented therapeutic landscape. Herein, the optimization pathways of existing therapeutics and emerging therapeutic directions are systematically reviewed, with particular emphasis on their mechanisms of action, current clinical application status, and future potential, with the aim of providing guidance for personalized IBD management.

### Refinement of current pharmacologic therapies: optimizing biologics and small-molecule agents

Pharmacological treatment of IBD has entered a phase of diversification and personalization, primarily consisting of biologics and small-molecule therapies. Since the late 1990 s, the variety of biologics has continuously increased, encompassing monoclonal antibodies targeting TNF-α, integrin inhibitors, and interleukin-12/23 inhibitors. These biologics have become the standard treatment for moderate to severe IBD patients, as well as for those who are refractory to conventional therapies, steroid-dependent, or steroid-resistant.

TNF-α is a critical pro-inflammatory cytokine that plays a central role in the pathogenesis of IBD. TNF-α activates multiple signaling pathways through binding to its receptors (TNFR1 and TNFR2), inducing the release of inflammatory mediators, cell proliferation, apoptosis, and immune cell recruitment, ultimately leading to chronic intestinal inflammation. Studies have shown that serum levels of TNF-α are significantly elevated in patients with CD and UC, with levels closely associated with disease activity, suggesting that TNF-α is a key driver of the inflammatory response in IBD [290]. Furthermore, TNF-α promotes the production of other pro-inflammatory cytokines, such as IL-1β and IL-6, further exacerbating the inflammatory response. To target this pathway, chimeric infliximab and fully human adalimumab can neutralize free TNF-α and induce apoptosis of TNF-α-expressing cells, significantly improving symptoms, promoting mucosal healing, and reducing surgery/hospitalization rates [291–294]. However, some patients experience primary or secondary failure due to anti-drug antibodies, insufficient drug levels, or individual differences in the immune environment. This has led to the concept of TDM, which involves quantifying drug concentrations and antibody levels to guide individualized dosing adjustments, thereby improving and maintaining long-term efficacy [295]. Regarding the safety of anti-TNF-α agents, skin reactions (such as eczematous and psoriatic rashes) are most common [296, 297]; while rare but severe neurotoxicities, such as Guillain-Barré syndrome and multiple sclerosis-like lesions, have also been reported [292, 298, 299]. Additionally, the rate of latent tuberculosis infections is relatively high, necessitating strict screening prior to treatment and, if necessary, adjustments to the treatment regimen [300]. Emerging therapeutic strategies, such as novel nanodelivery systems, are attempting to release drugs locally to reduce systemic exposure and minimize side effects [301, 302]. In conclusion, anti-TNF-α agents significantly improve prognosis by specifically blocking TNF-α and drive IBD towards precision medicine. Future efforts should integrate TDM, individualized dosing, and novel delivery technologies to achieve refined management.

Integrins are a class of cell surface receptors that are widely expressed on immune cells and are involved in multiple biological functions, including cell adhesion, signal transduction, and cell migration. In IBD, the integrin-mediated directed migration of leukocytes to the gut is considered a key event in the pathogenesis. Specifically, α4β7 integrin regulates the localization of lymphocytes to the gut mucosa by binding to the gut-specific cell adhesion molecule mucosal addressin cell adhesion molecule-1 (MAdCAM-1), promoting the accumulation of immune cells at the site of inflammation, thereby exacerbating the intestinal inflammatory response [303, 304]. Furthermore, it has been found that the expression of integrin β6 is significantly upregulated in regions of intestinal inflammation, and it plays a crucial role in regulating the intestinal immune environment, macrophage infiltration, and the integrity of the intestinal barrier. Deletion of this subunit alleviates inflammation and tissue damage in experimental colitis and related colorectal cancer models [305]. Therefore, blocking integrin-mediated leukocyte migration has become a key target in IBD drug development. Integrin inhibitors primarily target integrin subunits to block their interactions with cell adhesion molecules, reducing the migration of inflammatory cells to the gut and thereby alleviating intestinal inflammation. Vedolizumab (a humanized anti-α4β7 monoclonal antibody) exhibits gut selectivity [303, 304, 306], induces and maintains remission efficacy [307, 308], and is particularly suitable for patients who have failed anti-TNF therapy, with subcutaneous administration showing comparable efficacy and safety to intravenous delivery [309]; New drugs, such as etrolizumab (anti-β7), abrilumab (anti-α4β7-IgG2), PN-943 (oral peptide), AJM300(oral small molecule), and ontamalimab (anti-MAdCAM-1), further enhance target precision and drug delivery convenience [303, 306, 310]. Regarding safety, integrin inhibitors generally present a lower risk of infections and malignancies compared to anti-TNF therapy [311], However, case reports have indicated that vedolizumab may be associated with rare pulmonary inflammation (eosinophilic pneumonia) or virus-associated malignancies [312, 313]. Looking ahead, monitoring integrin drug concentrations, genetic risk scores, and intestinal immune phenotyping will enable integrin inhibitors to play a greater role in the personalized and precise treatment of moderate-to-severe UC/CD [314, 315].

IL, as a class of important cytokines, play a key role in the immunopathogenesis of IBD. IL-12 and IL-23 are two members of the IL-12 family, primarily secreted by antigen-presenting cells, and are involved in regulating intestinal immune balance and inflammatory responses. IL-12 mainly promotes the differentiation of Th1 cells and the production of interferon-γ, while IL-23 stimulates the expansion of Th17 cells and the secretion of pro-inflammatory cytokines, processes that collectively drive the inflammatory progression of IBD. IL-12 and IL-23 share a common p40 subunit, a characteristic that provides an important target for targeted therapy [288]. Biologics targeting IL-12/23 and IL-23 have recently become an important component of IBD treatment. Ustekinumab (anti-p40), targeting this pathway, induces and maintains remission in both CD and UC, with high steroid-free remission rates in children and adolescents at 26 and 52 weeks, and long-term safety profiles that are favorable [316]; Anti-IL-23p19 antibodies, such as risankizumab, guselkumab, brazikumab, and mirikizumab, have also demonstrated efficacy and favorable safety profiles [317, 318]. It should be noted that although IL-17 inhibitors are widely used in diseases such as psoriasis, they may induce or exacerbate IBD, and therefore are contraindicated in IBD patients [319–321]. This indirectly reflects that the application of biologics targeting different interleukins in IBD should be based on the disease characteristics and individual patient differences for precision therapy. Overall, anti-IL-12/23 and anti-IL-23 agents provide an effective new option for moderate-to-severe IBD in patients who fail or are intolerant to anti-TNF therapy, and future generations of anti-IL drugs targeting finer subunits or downstream signaling will further promote precision and personalized treatment.

Biologics have become the core therapy for moderate-to-severe IBD, significantly improving remission rates, promoting mucosal healing, and enhancing quality of life. Anti-TNF-α agents (infliximab, adalimumab) remain first-line options, significantly reducing intestinal inflammation by blocking TNF-α. However, approximately 30% of treatment-naïve patients are ineffective, and 20–40% experience secondary loss of response, requiring TDM and sequential therapy for refined management [322]. Vedolizumab reduces gut-specific lymphocyte migration. Studies such as VARSITY show that it has a higher endoscopic remission rate in UC compared to adalimumab, and similar efficacy in CD, making it suitable for patients with anti-TNF failure or intolerance [323]; Ustekinumab (anti-IL-12/23 p40) and tofacitinib (JAK inhibitor) offer new options for patients with multiple-line treatment failures [324]. Regarding safety, large cohort studies have confirmed that biologics are primarily associated with mild-to-moderate infections, with a low incidence of severe infections. The concomitant use of corticosteroids is significantly associated with infection risk; however, the infection risk of biologics such as anti-TNF and JAK inhibitors should be assessed in conjunction with individual patient characteristics [325]; COVID-19 data support that maintenance therapy during the pandemic does not increase the risk of severe illness [326]. Patient adherence directly impacts drug efficacy. The adherence to self-injection of adalimumab is superior to that of ustekinumab, potentially due to factors such as dosing intervals, injection methods, and storage conditions [327]; Anti-TNF-related rashes can be managed through continued use of the original drug, switching to a similar drug, or cross-class conversion, enabling personalized adjustment [328]. Cost-effectiveness analyses indicate that infliximab initiation therapy offers economic advantages in the UK/France, particularly for UC [329]. With the continuous emergence of novel biologics and small molecules, clinical decision-making must integrate disease conditions, medical history, comorbidities, and safety considerations to develop precise sequential strategies [330, 331]. In conclusion, the rational selection and sequential use of biologics, along with stringent infection monitoring and patient education, are key to maximizing efficacy and minimizing adverse reactions in moderate-to-severe IBD. Future head-to-head trials and real-world data will further optimize personalized treatment strategies.

Biologics have been used long-term in IBD, demonstrating overall safety and significant benefits; however, potential risks require stratified management. In the case of anti-TNF, the German BiKeR registry shows that 9 years of etanercept use in pediatric and adolescent rheumatoid arthritis patients did not observe new tumor or autoimmune risks during follow-up; however, the incidence of severe infections was higher compared to the control group that did not use biologics [332]; In the real world, infliximab and its biosimilars maintain long-term efficacy and safety consistent with the reference product [333, 334]. Data on new targeted therapies are even more promising: vedolizumab, as a “gut-specific” anti-integrin, has demonstrated a rare incidence of severe adverse events in registered trials, open extensions, and real-world data, while remaining safe in pregnant patients, children, the elderly, and those with a history of cancer [334]; The IL-23p19 inhibitor risankizumab also did not increase the risk of opportunistic infections or enteritis [335]. Advanced combination therapy(two biologics or biologic + small molecule) can improve remission rates in refractory cases; however, long-term randomized controlled evidence is lacking, and safety requires continued validation [336, 337]. In elderly patients, the hospitalization rate for infections with vedolizumab or ustekinumab is lower than with anti-TNF, and the overall adverse event rates are similar [338, 339]; Organ transplant recipients using biologics must be cautious of severe infections, but no significant difference in tumor risk has been observed [340, 341]. In terms of immunogenicity, the efficacy of anti-TNF is more commonly reduced due to the formation of anti-drug antibodies (ADA), while the incidence of ADA in newer generations of anti-integrins and anti-interleukins is lower, providing a basis for optimizing sequential therapy [342]. Biosimilars and reference products show no difference in long-term efficacy or safety, and can reduce costs and increase accessibility [334, 343]. Despite the overall good safety profile, attention must still be given to risks such as infections(including herpes zoster), skin cancer, lymphoma, and infusion/injection reactions [344, 345]; In long-term follow-up, the concomitant use of corticosteroids significantly increases the risk of infections [346], and very rare cases of multiple immune modulation-related malignancies have also been reported [341]. Current long-term safety studies of biologics have limitations, including selection bias in clinical trials, limited sample sizes and follow-up durations, lack of standardized safety outcome definitions, and heterogeneity in real-world data. Future research should focus on large-scale, long-term, multi-center cohort studies and randomized controlled trials, integrating biomarkers and genetic information to achieve personalized risk assessment and precision therapy. Furthermore, safety studies in specific populations(such as children, elderly individuals, organ transplant recipients, and pregnant women) need to be strengthened to guide rational clinical prescribing.

Janus kinases (JAKs) are a class of intracellular tyrosine kinases, including four subtypes: JAK1, JAK2, JAK3, and tyrosine kinase 2 (TYK2). They are involved in the signaling of various cytokine receptors and are key components of the JAK-STAT(signal transducer and activator of transcription) pathway. This pathway regulates the differentiation, proliferation, and inflammatory response of immune cells and plays a crucial role in the pathogenesis of IBD. The abnormal expression of various pro-inflammatory cytokines in the body of IBD patients triggers intestinal inflammation through the JAK-STAT pathway. JAK inhibitors, as small-molecule drugs, block the tyrosine kinase activity of JAK family members, interrupt cytokine signaling, and inhibit downstream signals of pro-inflammatory cytokines, ultimately reducing intestinal inflammation [341, 347]. By blocking the kinase activity, JAK inhibitors rapidly suppress inflammation through multiple targets, with oral administration that is non-immunogenic, thus becoming a novel strategy in small-molecule therapies [348–350]. Tofacitinib is a non-selective JAK inhibitor (targeting JAK1/2/3/TYK2) that has been approved by the food and drug administration (FDA) for the induction and maintenance of remission in moderate to severe UC, particularly in patients who have failed traditional immunomodulators or biologic agents [348, 351]; It has a fast onset of action with oral administration and exhibits the distinct advantage of being free from anti-drug antibodies [352]. However, its efficacy in CD is limited, and it has not been approved for this indication, potentially due to pathway differences [348, 353]. The next-generation selective JAK1 inhibitors, upadacitinib and filgotinib, demonstrate good efficacy in both CD and UC by precisely targeting JAK1. Upadacitinib was approved by the EU and FDA in 2023 for moderate to severe CD, expanding the oral small-molecule options [348, 354, 355]. Regarding safety, common adverse events of tofacitinib include herpes zoster infections, hypercholesterolemia, venous thromboembolism (VTE), and cardiovascular events. The risk significantly increases in individuals aged ≥ 50 years with cardiovascular risk factors, necessitating strict risk stratification and monitoring [348, 356–359]. Real-world data indicate that the incidence of serious infections and thrombosis with tofacitinib is relatively low; however, early identification and personalized management remain necessary [360, 361]; Its short half-life and oral administration advantage also facilitate bridging or switching after biologic failure [362, 363].

### Emerging therapies: novel mechanistic targets arising from pathobiology

In recent years, significant advancements have been achieved in the treatment of IBD, particularly with the introduction of biologic agents, which have substantially improved disease management. However, despite the widespread use of current therapeutic strategies, both primary and secondary treatment failure rates remain high, indicating that existing therapies continue to have limitations in efficacy and safety, thereby driving the development of new therapeutic targets and approaches.

#### Emerging targets related to immune regulation

Tregs are central to maintaining intestinal immune homeostasis, as they prevent autoimmunity and chronic inflammation by suppressing excessive immune responses, serving as a key safeguard for intestinal immune tolerance. In patients with IBD, deficits in both the number and function of Tregs lead to immune dysregulation and exacerbation of inflammation [363]. The stability and suppressive function of Tregs are regulated by multiple factors, including Foxp3, IL-2 signaling, and metabolic state [363–365]. Therefore, enhancing both the number and function of Tregs has become an important therapeutic strategy in the treatment of IBD. Recently, various novel strategies aimed at promoting Treg expansion and function have emerged. Low-dose IL-2 selectively expands Tregs without activating effector T cells, and has been widely studied [366]. Gut microbiota metabolites, such as SCFAs and isoalloLCA, enhance Treg function by upregulating Foxp3 [367, 368]. At the metabolic level, maintaining mitochondrial function is crucial for Treg stability, and inhibiting IL-21-induced metabolic reprogramming can restore their suppressive capacity [364]. Immune checkpoint molecules, such as cytotoxic T-lymphocyte-associated protein 4 (CTLA-4) and programmed death-1 (PD-1), play crucial roles by downregulating co-stimulatory signals [369] and maintaining Treg stability [370]. α1,3-fucosyltransferase VII (FUT7) enhances the suppressive function of Tregs by promoting intestinal homing and downregulating PD-1 expression, showing potential value in IBD treatment [371]. In clinical treatment, optimized autologous Tregs expanded in vitro have shown potential to improve IBD inflammation [372]. Chimeric antigen receptor (CAR) technology confers intestinal antigen recognition capability to Tregs for directed regulation [373] Exosomes, such as isoalloLCA mediators, can slow IBD progression by inhibiting the NF-κB pathway [367]. Overall, IBD therapeutic strategies that enhance Treg function encompass multiple dimensions, including cytokine regulation, metabolic modulation, immune checkpoint activity, and cell/exosome therapies. In the future, the combination of gene editing, metabolic regulation, and targeted delivery technologies is expected to further enhance Treg efficacy, offering precise and effective treatment for IBD patients.

Chronic intestinal inflammation in IBD is closely associated with key innate immune receptors (NOD2, TLR). NOD2 regulates NF-κB/MAPK through the receptor-interacting serine/threonine-protein kinase 2 (RIPK2)- X-linked inhibitor of apoptosis protein(XIAP) axis, and drugs targeting this interaction have shown potential for alleviation [374, 375]; Abnormal expression of receptors such as TLR3, TLR5, TLR7, and TLR8 disrupts the barrier and amplifies inflammation [376]. Animal models and clinical validation: TLR blockers reduce inflammatory mediators [377]; NLRP3 inhibitors significantly improve experimental colitis by reducing the secretion of IL-1β and IL-18 [378]. Other studies have shown that certain dietary ligands can influence innate immune responses and reduce intestinal inflammation by modulating nuclear receptors such as peroxisome proliferator-activated receptors (PPARs) [379]. Traditional therapies, such as acupuncture, have also been shown to modulate TLR and nucleotide-binding oligomerization domain-like receptor (NLR)-associated signaling pathways to achieve immune modulation [380]. At the mechanistic level, RNA-binding protein QKI regulates macrophage polarization, and TLR4 single nucleotide polymorphisms (SNPs) reveal genetic susceptibility [381], providing molecular and genetic foundations for precise intervention. Overall, the NOD2 and TLR families are central regulatory nodes in the innate immune response of IBD; in the future, the combination of specific receptor modulators, molecular targeting, and immune regulation is expected to achieve personalized treatment and precise inflammation control.

#### Repair and protection of intestinal barrier function

TJs play a critical role in maintaining the integrity of the intestinal barrier. Recently, several novel therapeutic strategies targeting the regulation of tight junction protein expression and function have emerged. Natural products, such as chitosan oligosaccharides (COS), upregulate tight junction protein expression and improve intestinal barrier function by activating the ERK1/2 signaling pathway [382]; Cedrol alleviates inflammation-induced barrier dysfunction by promoting ATP synthesis and enhancing tight junction protein mRNA expression [383]. Plant polyphenols, such as huperzine A and its metabolites, inhibit LPS-induced MAPK phosphorylation, protect tight junction proteins, and maintain intestinal permeability [384]. Probiotic products, such as Limosilactobacillus reuteri, enhance the expression of heat shock proteins(heat shock protein 70 (HSP70), HSP25), promote tight junction protein synthesis, and alleviate inflammation-induced intestinal barrier damage [385]. Intervening in inflammatory signaling pathways is also a key strategy. Menstrual blood-derived endometrial mesenchymal stem cells (MenSCs) inhibit the NF-κB/Snail axis, reduce inflammatory factor expression, promote tight junction protein recovery, and repair the barrier [386]. Statin molecules activate the PI3K-Akt signaling pathway, promoting tight junction protein expression and epithelial repair [387]. Additionally, traditional Chinese medicine formulas, such as Yuyang Decoction, regulate the TLRs/Tollip signaling pathway to promote tight junction protein repair and alleviate colitis [388]. Emerging gene therapies and targets have also attracted attention. For example, defects in the protein tyrosine phosphatase, non-receptor type 2 (PTPN2) gene lead to abnormal claudin-2 expression, impairing barrier function, which can be partially restored by administering recombinant protease inhibitors [72, 389]. Furthermore, the cholesterol-metabolizing enzyme cholesterol 25-hydroxylase (CH25H) and its product, 25-hydroxycholesterol, regulate activating transcription factor 3 (ATF3) expression to promote tight junction protein production and protect the intestinal barrier [390]. A high-cholesterol diet can inhibit sterol regulatory element-binding protein 2 (SREBP2), promoting the endocytic degradation of tight junction proteins and worsening barrier function, highlighting the importance of metabolic regulation in maintaining the intestinal barrier [391]. TJs are central to maintaining the integrity of the intestinal barrier. Multiple strategies, including anti-inflammatory, antioxidant, metabolic, stem cell, and gene interventions, synergistically upregulate TJs expression and function, providing diverse molecular targets and clinical pathways for the precise repair of barriers in IBD.

In recent years, research has focused on the signaling pathways regulating intestinal epithelial cell regeneration and apoptosis, particularly the canonical Wnt/β-catenin pathway, which plays a crucial role in the proliferation and differentiation of intestinal epithelial cells. The Wnt/β-catenin signaling pathway regulates the self-renewal and fate determination of Lgr5 + intestinal stem cells, promoting the rapid regeneration of the intestinal epithelium. A series of studies have shown that dysregulation of Wnt signaling in the intestines of IBD patients and DSS-induced mouse models impedes epithelial cell regeneration, exacerbating intestinal inflammation and barrier disruption. Furthermore, the regulation of Wnt signaling not only affects stem cell proliferation but also directs the differentiation of epithelial cells, maintaining the balance of various cell types, such as goblet cells and enteroendocrine cells, thereby preserving the integrity of the intestinal barrier [392]. Moreover, multiple signaling pathways are involved in regulating intestinal epithelial cell apoptosis, leading to barrier disruption. Activation of the AKT signaling pathway inhibits apoptosis and maintains cell survival. 3-mercaptopyruvate sulfurtransferase (MPST) modulates AKT signaling, protecting intestinal epithelial cells from apoptosis and alleviating IBD pathology [393]. Additionally, pleckstrin homology-like domain family a member 1 (PHLDA1) protects barrier function by limiting apoptosis of intestinal epithelial cells. Its loss promotes apoptosis and exacerbates colitis [394]. Parkinsonism-associated deglycase (DJ-1) has also been shown to have anti-apoptotic effects. The loss of DJ-1 promotes p53-mediated intestinal epithelial cell apoptosis, worsening colitis [395]. Therapeutic strategies targeting these molecular targets, such as the incorporation of the p53 inhibitor pifithrin-α into nanoparticle formulations, are expected to improve IBD symptoms by precisely inhibiting excessive apoptosis [396]. In recent years, the roles of microRNAs and circular RNAs in regulating intestinal epithelial cell proliferation and apoptosis have gradually been revealed. miR-7 regulates the EGFR/NF-κB/AKT/ERK signaling axis to control the proliferation of intestinal epithelial cells and the secretion of inflammatory cytokines. The loss of miR-7 promotes epithelial cell regeneration and immune modulation, alleviating colitis [397]. circHIPK3, a circular RNA, promotes intestinal epithelial cell repair and regeneration by sequestering miR-29b and regulating factors such as Rac1, Cdc42, and Cyclin B1 [398]. Additionally, miR-31 exhibits a “spring effect” balancing inflammation and regenerative responses at different stages of inflammation [399]. On the therapeutic front, several emerging therapies have been shown to promote intestinal epithelial cell regeneration. Human adipose-derived mesenchymal stem cell exosomes(hADSC-Exo) promote the proliferation of Lgr5 + intestinal stem cells and epithelial cells, repairing the intestinal barrier [400]. Natural products, such as black tea polyphenols and walnut oil, alleviate intestinal epithelial cell inflammation and apoptosis by regulating the TLR4/MyD88/NF-κB pathway [401]. Additionally, recombinant soluble thrombomodulin (rsTM) has been shown to promote the proliferation of intestinal stem cells and facilitate mucosal repair in DSS model mice [402]. Specific Wnt signaling agonists, such as FZD5/8 and the LRP6 receptor-specific Wnt mimic SZN-1326-p, have been found to promote the repair of damaged intestinal epithelium and alleviate inflammation [403]. Traditional Chinese medicine formulations, such as Sha Yao Tang and Wu Wei Wan, promote the regeneration of intestinal epithelial stem cells and the repair of the mucosal barrier through multi-target regulation, demonstrating considerable therapeutic potential [404, 405]. Regarding the relationship between intestinal epithelial cell metabolism and repair, studies indicate that metabolic reprogramming of these cells affects their regenerative capacity. Changes in fatty acid metabolism, mitochondrial function, and energy metabolism all influence cell proliferation and differentiation [406, 407], Additionally, certain microbiota-derived metabolites, such as butyrate, support the energy metabolism and homeostasis of intestinal epithelium [396]. Furthermore, the regulation of calcium signaling pathways in intestinal epithelial cells is closely related to regeneration. For example, cholinergic neurons stimulate Ca2 + currents in intestinal epithelial cells, promoting their maturation and repair [408].

The mucosal layer is a key component of the intestinal barrier system, and emerging therapies for its protection focus on a three-step strategy of “thickening—augmentation—quality enhancement”: First, orally administered ROS-responsive thiolated hyaluronic acid hydrogel (HASH) has been shown to form an artificial mucous-like layer at the lesion site [409], physically blocking pathogen invasion and remodeling the microbiota; Secondly, the plant compound arctigenin inhibits mitochondrial apoptosis in goblet cells, thereby increasing their number and promoting the integrity of the mucosal barrier [410], Simultaneously, IL-22 is used to stimulate the expansion of intestinal epithelial stem cells and upregulate the expression of membrane-bound mucins [411], thereby enhancing endogenous mucin secretion and component quality; Finally, miRNA regulation is employed to further fine-tune the secretory function of goblet cells and the structure of mucins [412], collaboratively reconstructing the mucosal barrier and restoring intestinal homeostasis.

#### Application of probiotics and prebiotics

Probiotics and prebiotics, as effective means of regulating gut microbiota balance, have demonstrated significant potential in the treatment of IBD. From a clinical perspective, probiotics have shown certain efficacy in the treatment of UC patients. For example, Escherichia coli Nissle 1917 and the multistrain formulation VSL3 have been confirmed to induce and maintain remission in UC [413, 414]. hese probiotics exert their effects through various mechanisms, including the inhibition of pathogenic bacteria growth, repair of the intestinal barrier, modulation of immune responses, and reduction of oxidative stress [414, 415]. However, the clinical efficacy of probiotics in CD patients remains unclear, with inconsistent results from multiple clinical trials, and they cannot yet be widely recommended [413, 416]. Prebiotics, such as oligosaccharides, fructooligosaccharides, and inulin, serve as “food” for probiotics, promoting their growth and metabolism, enhancing SCFA production, and indirectly exerting anti-inflammatory effects [417, 418]. The combined use of probiotics and prebiotics has shown a synergistic effect in clinical settings, effectively regulating gut microbiota and improving symptoms and quality of life in IBD patients [419, 420]. In recent years, genetically engineered probiotics have emerged as next-generation therapies. Through synthetic biology techniques, probiotics are modified to enable targeted release of therapeutic molecules in the gut, enhancing anti-inflammatory effects and improving colonization ability [415, 421, 422]. For example, immune regulatory molecules were bioorthogonally conjugated to Escherichia coli Nissle 1917, combined with enteric coatings, achieving immune polarization of inflammatory macrophages and significantly alleviating colitis in mice [423]. In addition, the development of intelligent microcapsule delivery systems, such as microsphere gels coated with hyaluronic acid and catechins, can protect engineered probiotics from gastric acid environments, prolong intestinal colonization time, and enhance therapeutic efficacy [422]. These technological advancements offer new possibilities for the precise treatment of probiotics, particularly showing promising prospects in the field of personalized medicine [424]. Overall, probiotics and prebiotics exert multiple roles in the treatment of IBD, including restoring microbiota balance, modulating immune responses, and repairing the intestinal barrier. Clinical efficacy is more established in UC patients, while the therapeutic effects in CD patients require further validation. The emergence of novel genetically engineered probiotics provides innovative strategies for the treatment of IBD; however, further high-quality clinical trials are needed to standardize dosage, strain selection, and safety evaluations, facilitating the translational application of these therapies. In the future, research on the combined use of probiotics and prebiotics should be strengthened, incorporating molecular biology and microbiology to promote the development of precise, personalized IBD treatment strategies.

#### Application of fecal microbiota transplantation

Fecal microbiota transplantation (FMT) involves the transfer of the gut microbiota from a healthy donor to the patient's intestine in order to restore intestinal microbial balance. This technique was initially widely used due to its effective therapeutic outcomes in recurrent clostridium difficile infection (CDI) and has more recently been explored for the treatment of IBD. FMT can be performed through various routes, including colonoscopy, enemas, nasogastric tubes, or oral capsules. Among these, colonoscopy and enemas deliver the microbiota directly to the colon, while oral capsules are of particular interest due to their ease of administration and high patient compliance. Studies suggest that using frozen fecal matter and minimizing the donor-recipient time interval may further enhance therapeutic efficacy [425]. In the treatment of IBD, FMT has demonstrated potential to alleviate symptoms and improve endoscopic findings by restoring gut microbiota diversity and modulating immune responses. Several randomized controlled trials (RCTs) have confirmed its effectiveness in inducing remission in mild-to-moderate UC [426, 427]; Although FMT has also been attempted in CD and refractory IBD, reports on its efficacy have been inconsistent [428], These discrepancies may be attributed to factors such as disease heterogeneity, donor selection, and transplant protocols. In terms of safety, FMT is generally considered a safe treatment. Common adverse effects include mild gastrointestinal discomfort, such as diarrhea, bloating, and nausea, which are typically transient and reversible [429, 430]. However, the unique immune status and compromised intestinal barrier in IBD patients may increase the risk of infection and potentially exacerbate disease activity. Some studies have reported a few severe adverse events, emphasizing the importance of stringent donor screening and standardized procedures [431, 432]. Future optimization primarily includes the standardization of FMT procedures, such as donor screening and management, fecal preparation and storage, and the determination of administration routes and doses [425, 433]. The development of personalized treatment plans has also gained increasing attention, incorporating recipient microbiome characteristics and disease subtypes, and employing advanced technologies such as machine learning to predict FMT efficacy, thereby enabling precision microbiota-based therapy [434, 435]. Additionally, innovative strategies such as synthetic microbiota (SynCom) and probiotic pretreatment are being explored to enhance colonization rates and efficacy [259, 436]. Long-term follow-up studies are equally crucial for evaluating the durability of FMT efficacy and safety [437].

#### Targeted therapy of metabolic pathways

SCFAs are the primary metabolic products produced by gut microbiota through the fermentation of dietary fibers, with acetate, propionate, and butyrate being the most critical. These compounds play significant roles in the pathogenesis and treatment of IBD. Butyrate, as a key member of SCFAs, not only serves as the primary energy source for intestinal epithelial cells but also regulates gut immune responses and barrier function through multiple pathways, thereby maintaining intestinal homeostasis. Butyrate promotes the repair and maintenance of the intestinal mucosal barrier, enhancing the expression of tight junction proteins such as Occludin, thereby reducing intestinal permeability and preventing the invasion of pathogenic microorganisms and harmful substances [438]. Additionally, butyrate regulates gut immune cell function by inhibiting histone deacetylase (HDAC) activity, promoting the differentiation of Treg, and suppressing the release of pro-inflammatory cytokines such as IL-6 and TNF-α, thereby alleviating intestinal inflammation [439, 440]. Studies have shown that butyrate enhances the expression of granzyme B in IL-10-producing Th1 cells, maintaining immune tolerance and preventing excessive inflammatory responses [441]. By modulating the gut microbiome, butyrate also promotes the proliferation of beneficial bacteria such as Roseburia intestinalis, which has the ability to produce butyrate. A reduction in its abundance is closely associated with the exacerbation of IBD [442, 443]. Natural polysaccharides and other substances can promote the production of SCFAs, thereby exerting anti-inflammatory effects [444, 445]. Additionally, SCFAs can mediate immune modulation and intestinal barrier protection by binding to G protein-coupled receptors [441]. Therapeutic strategies targeting the regulation of metabolite levels in IBD have garnered increasing attention. For example, the use of prebiotics, probiotics, or their metabolites that promote SCFA production has been shown to improve gut microbiota, enhance barrier function, and regulate inflammatory responses [438, 446]. Additionally, functional molecular coatings such as GelNB can form a biophysical barrier, isolating intestinal-stimulating metabolites and effectively protecting the intestinal mucosa [447]. Natural products such as the flavonoid compound Xanthohumol regulate the gut microbiota and its metabolites, improving IBD-related osteoporosis and intestinal inflammation [448]. Polysaccharides, such as glycyrrhizin lysine, promote SCFA production, regulate the Th17/Treg balance, and alleviate colitis and associated depressive symptoms [449]. Additionally, adjustments to the dietary structure, such as the supplementation of soluble dietary fibers, significantly increase SCFA levels and improve immune function [450, 451].

Cellular energy metabolism regulation plays a crucial role in the pathogenesis of IBD, particularly during the metabolic reprogramming of intestinal immune cells. Intestinal immune cells, such as macrophages and T cells, undergo significant changes in metabolic pathways during inflammatory states, which not only affect their functional status but also determine the nature and intensity of the immune response. In the IBD inflammatory microenvironment, macrophages polarize to the M1 phenotype, characterized by enhanced glycolysis, accompanied by a reduction in pyruvate dehydrogenase (PDH) activity, leading to lactate accumulation and a shift in energy supply mechanisms. This metabolic reprogramming promotes the expression of pro-inflammatory cytokines and exacerbates the inflammatory response. Studies show that thiamine deficiency further inhibits PDH activity, accelerates M1 polarization, and exacerbates experimental colitis [452], while lactate dehydrogenase inhibitors can block this vicious cycle and partially restore immune homeostasis [452]. Meanwhile, the metabolic switch involving mTORC1 inhibition and AMP-activated protein kinase (AMPK) activation in macrophages has been shown to downregulate the aforementioned inflammatory factors, revealing a close link between metabolic signaling pathways and immune inflammation [453], providing dual molecular targets for targeting glycolysis in IBD. The core of targeting glycolysis lies in regulating the metabolic state of macrophages to alter their functional polarization, thereby alleviating the inflammatory response. PDH activators or lactate dehydrogenase inhibitors can block excessive glycolysis in macrophages, limiting M1 pro-inflammatory polarization and alleviating intestinal inflammation. Meanwhile, AMPK, as an energy sensor, its activation not only suppresses inflammatory signaling but also induces autophagy and antioxidant responses, working synergistically to improve barrier function [453, 454]. On the other hand, fatty acid oxidation (FAO) is a key metabolic switch driving M2 anti-inflammatory polarization. Retinol saturated enzyme (RetSat) deficiency upregulates FAO, reducing oxidative stress and repairing colonic epithelial structure, confirming that regulating lipid metabolism can exert therapeutic value for IBD from a dual “immune-epithelial” perspective [455]. The restoration of mitochondrial function and energy metabolism is a core element in IBD inflammation regulation. Mitochondrial damage causes ATP depletion and ROS burst, exacerbating inflammation and disrupting the intestinal barrier. Numerous studies have pointed out that strategies targeting mitochondrial metabolism, such as CoQ10 supplementation and promoting mitochondrial autophagy, can effectively improve the pathological state of IBD and alleviate intestinal inflammation [456, 457]; At the same time, NAD + metabolism remodeling has also become a new focus. By supplementing NAD + precursors or activating the Sirtuin 1 (SIRT1)/AMPK pathway, mitochondrial biogenesis and antioxidant capacity can be synergistically enhanced, correcting immune-metabolic imbalance in both directions and providing an additional molecular target for IBD intervention [458, 459].

#### Targeting of signaling molecules and their receptors

RIPK2 serves as a critical mediator in the NOD1/2 signaling pathway, playing a significant role in the pathogenesis and progression of IBD. Studies have shown that the expression of RIPK2 is significantly upregulated in the intestinal mucosa of IBD patients, promoting the release of inflammatory factors and thereby exacerbating local inflammatory responses. It has been pointed out that the interaction between RIPK2 and XIAP is a key target for regulating NOD1/2-dependent immune responses, and the potential for drug development targeting this protein–protein interaction is substantial [374]. In several animal models, inhibition of RIPK2 kinase activity significantly alleviated colonic inflammation. Oral administration of the RIPK2 inhibitor BI 706039 in the tumor necrosis factor receptor 1 (TNFR1)-related unfolded protein response (UPR)-mediated colitis (TRUC) mouse model of spontaneous colitis significantly reduced histopathological inflammation scores in colonic tissues and related inflammatory markers, demonstrating the effectiveness of RIPK2 inhibitors in modulating intestinal immune system signaling [460]. Furthermore, novel RIPK2 inhibitors such as Zharp2-1 and HYML-122 have demonstrated favorable pharmacokinetic properties and the ability to inhibit NOD-mediated NF-κB/MAPK activation. These inhibitors have been shown to effectively alleviate inflammation in both mouse and rat colitis models and significantly suppress the release of pro-inflammatory cytokines in clinical samples, highlighting their potential as candidate drugs for IBD treatment [461, 462]. From a molecular mechanism perspective, RIPK2-mediated signaling activates the NF-κB and MAPK pathways, promoting the release of pro-inflammatory cytokines such as TNF-α, IL-6, and IL-12/23, thereby driving the inflammatory response. Aberrant activation of RIPK2 is closely associated with increased expression of signaling molecules in the colonic mucosa of IBD patients, and blocking RIPK2 activation effectively inhibits the development of experimental colitis in mice [463, 464]. Notably, dual inhibitors targeting RIPK2 (simultaneously inhibiting RIPK2 and RIPK3) have also been found to effectively suppress NOD-induced cytokine production and necroptotic cell death. In a DSS-induced mouse model of colitis, these inhibitors demonstrated significant therapeutic efficacy without noticeable toxicity, suggesting that dual inhibition strategies may offer new perspectives for IBD treatment [465]. Moreover, studies have also revealed that RIPK2 regulation involves various enzymes with ubiquitination modifications, such as deubiquitinases OTUB2 and YOD1, which stabilize RIPK2 and enhance NOD2 signaling, promoting protective intestinal immune responses. Conversely, NEDD4 binding protein 3 (N4BP3) exacerbates the inflammatory response by facilitating K63-linked ubiquitination of RIPK2, suggesting that regulating RIPK2 ubiquitination status may also serve as a potential therapeutic target [466–468].

The farnesoid X receptor (FXR) agonists demonstrate significant potential in alleviating intestinal inflammation and improving gut barrier function in IBD. As a nuclear receptor, FXR primarily regulates bile acid metabolism, intestinal homeostasis, and immune modulation. Its activation has been shown to suppress intestinal inflammation and promote the repair of the gut barrier [469]. For example, synthetic FXR agonists, such as compound 33, exhibit strong anti-inflammatory activity and the ability to repair colonic epithelium in the DSS-induced acute colitis model, suggesting their potential as candidate drugs for IBD treatment [470]. Moreover, the human intestinal fungus Candida metapsilosis M2006B significantly mitigates murine colitis by secreting novel FXR agonists, further supporting the therapeutic potential of FXR agonists in IBD [471]. Additionally, FXR agonists exert protective effects through multiple pathways. GW4064, an FXR agonist, inhibits necroptosis of intestinal epithelial cells induced by inflammatory factors, thereby maintaining the integrity of the intestinal barrier [472]. Furthermore, FXR activation regulates intestinal immune cells, particularly innate ILCs, suppresses the expression of pro-inflammatory cytokines, and modulates immune homeostasis [473]. In preclinical studies, FXR agonists, such as obeticholic acid, have been approved for the treatment of primary biliary cholangitis and have shown promising therapeutic potential in intestinal diseases like IBD, although their side effects and tissue specificity require further optimization [474, 475]. FXR agonists can also influence the gut microbiota composition. The FXR agonist fexaramine not only restores intestinal FXR activity in mice induced by deoxycholic acid(DCA), but also regulates the abundance of short-chain fatty acid-producing bacteria, thereby alleviating intestinal inflammation [476]. Furthermore, exosomes derived from mesenchymal stem cells (MSCs) inhibit macrophage inflammation by activating the SIRT1-FXR signaling pathway and reduce NLRP3 inflammasome activation, thereby alleviating IBD inflammation [477, 478]. The direct use of synthetic FXR agonists presents off-target effects and limited efficacy, making exosome-mediated FXR signaling regulation an innovative and effective therapeutic strategy. MSC-Exosomes enriched with miRNA and proteins reduce FXR acetylation through the SIRT1-FXR axis, block NLRP3 inflammasome activation, and rapidly suppress excessive inflammation in intestinal macrophages [477]; The same exosomes also remodel the microbiota-metabolite network, upregulate FXR expression, restore barrier integrity, and reduce colitis inflammation [478], thus linking “immune suppression” with “microecological repair” into a closed-loop. The FXR agonist-loaded MSC-Exosomes were further validated in the liver fibrosis model for their “drug-controlled release + targeted homing” functionality. This approach enhances bile acid signaling regulation efficiency while avoiding off-target toxicity [479]; Even exosomes derived from camel milk can reduce oxidative stress and restore intestinal barrier integrity through the FXR/NF-κB pathway [480], suggesting that exosomes derived from plants, animals, and humans can serve as “portable activators” of FXR signaling.

S1P is a bioactive sphingolipid molecule that regulates various cellular functions, including cell survival, differentiation, migration, proliferation, and immune responses, through its binding to five G protein-coupled receptors (S1PR1 to S1PR5) [69]. Modulators targeting S1P receptors have emerged as a promising approach for the treatment of IBD. Modulating S1P receptors, particularly S1PR1, effectively inhibits lymphocyte migration to inflamed regions, thereby alleviating intestinal inflammation [481]. S1PR modulators such as ozanimod and etrasimod, targeting this axis, induce S1PR1 internalization and degradation, blocking lymphocyte sensing of the S1P gradient, thereby reducing lymphocyte accumulation in peripheral blood and intestinal tissues. These agents have demonstrated good efficacy and oral convenience in inducing and maintaining remission in UC, as confirmed in multiple Phase III trials [482, 483]; However, the initial dose may cause transient bradycardia or atrioventricular block, and S1PR2/3 activation may increase cardiovascular risk. Therefore, a thorough assessment of the cardiac baseline and continuous ECG monitoring are required prior to administration [484, 485].

#### Targeted therapy against ROS

A synthetic antioxidant regimen has been established with four main approaches: “lipid-lowering, blood pressure-lowering, thiol supplementation, and enzyme replacement” Statins block the mevalonate pathway by inhibiting 3-hydroxy-3-methylglutaryl-coenzyme A (HMG-CoA) reductase, reduce Rac1 membrane translocation, thereby lowering ROS derived from NOX, while stabilizing eNOS mRNA, increasing NO bioavailability, and inhibiting various adhesion molecules, achieving a dual suppression of “oxidative stress and inflammation” [486]. ACE inhibitors (ACEI) reduce the generation of Ang II and block oxidation and fibrosis signaling driven by NF-κB. N-acetylcysteine (NAC) is rapidly converted into glutathione (GSH) in the small intestine [487], directly scavenging H₂O₂/O_2_•^−^ and inhibiting the NF-κB-iNOS axis [488], thereby remodeling the interaction between NOX2/iNOS/myeloperoxidase (MPO). Chemical modification of superoxide dismutase (SOD) with low molecular heparin [489], phospholipids [490, 491], mannosylation [492], or cationization [493] significantly extends its half-life and enables membrane-targeting ability. Recombinant TtSOD [494] and MS-AOE [495] exhibit acid and protease resistance in the TNBS/5-fluorouracil (5-FU) model.

Phospholipase C (PLC) chelates Fenton iron [496] and inhibits NOX4, with clinical rectal irrigation for 14 days leading to a reduction in the activity index by 80% in patients with mild-to-moderate UC [497]. Myeloperoxidase (MPO) inhibitors (4-MeO-TEMPO [498], ABAH [499, 500], SDG [501–505], KYC [506]) reduce HOCl generation and activate the Nrf2- heme oxygenase-1(HO-1) axis.

Natural antioxidants form a multidimensional “polyphenol-terpene-alkaloid-monoterpene” network: Triterpenic acids(ursolic acid, oleanolic acid) inhibit the mitochondrial ROS-NF-κB-NLRP3 axis [507], restoring the Th17/Treg balance [508] and increasing tight junction proteins; Thymol activates Nrf2-SOD/GPx [509], inhibits cyclooxygenase-2 (COX-2) [510], and promotes Lactobacillus abundance [509]; Anthocyanins scavenge OH• more efficiently than vitamin C/E (Vit C/E), upregulate Nrf2-HO-1 [511], and improve microbial diversity [512], while high-dose purple corn diet further reduces inflammatory markers during the infliximab (IFX) maintenance phase [513]; Resveratrol donates hydrogen to scavenge ROS, upregulates SIRT1-Nrf2 [514], and inhibits the IL-6/STAT3 fibrosis axis [515, 516]. Curcumin scavenge ROS, inhibits IKKβ-S-nitrosylation [517], downregulates NLRP3 [518]/STAT3 [519], and regulates follicular helper T cell (Tfh) subsets [520, 521]. A meta-analysis demonstrates that its addition to 5-aminosalicylic acid (5-ASA) improves clinical remission rates in UC, although the endoscopic benefit remains highly heterogeneous [522–524]. The latest meta-analysis (Explore 2024, 8 RCTs, *n* = 482) confirms a clinical relative risk of approximately 2.33, while endoscopic remission is not significant(I^2^ = 69%) [523]. A 3 g/day crystalline formulation for 4 weeks achieved clinical and endoscopic remission in 65% of patients [525], compared to 20% with placebo, while the low-dose 450 mg/day was ineffective [525]. The micellar formulation (1.8 g/day) increased the target achievement rate by 1.8 times [526], suggesting that doses ≥ 2 g/day and high bioavailability formulations are key to breakthrough. Maintenance therapy with 2 g/day + 5-ASA for 6 months reduced the relapse rate from 20% to 5%, though the sample size was small [527]. Quercetin chelates metals and disrupts the STAT1/PPARγ balance, promoting M2 polarization [528]; nanodelivery systems are addressing its solubility issues [529, 530]. Catechins activate SOD [531]/GPx [532] and inhibit NF-κB [531, 532], completely inhibiting TNBS-induced colonic adhesions [533]. Silymarin downregulates MPO/MDA [534] and blocks the IL-6/STAT3 [535]-cell division cycle 25C (Cdc25C)/(cyclin-dependent kinase 1) CDK1 [536] proliferation signal; nanoparticles improve its water solubility [115, 537, 538]. Berberine directly scavenges ROS and inhibits NOX, activating the bitter receptor-Tuft-IL-25-ILC2-IL-13 repair axis [539] and remodeling the microbiota [540]. Sulforaphane activates Nrf2-antioxidant response element (ARE) [541] and promotes M2 polarization [542], also exhibiting anti-Helicobacter pylori activity [543].

Trace micronutrients form a “metal-vitamin” synergistic network: Vit C directly reduces O_2_•^−^/OH•/H₂O₂ and regenerates GPx and Vit E, activating Nrf2 [544] while inhibiting NF-κB-NLRP3 [544], which increases Occludin/Claudin-1/ZO-1 [545]. Vit D induces SOD, catalase (CAT) and GSH [546] and activates klotho protein(Klotho)-Nrf2 [547]. Vit E phenolic hydroxyl groups interrupt lipid peroxidation chains, increasing GPx/SOD [548] and blocking TGF-β1- Phosphorylated extracellular signal-regulated kinase(pERK), phosphorylated extracellular signal-regulated kinase (pSmad2), and phosphorylated c-Jun N-terminal kinase (pJNK) fibrosis signaling [549], while increasing Roseburia abundance [550]. Selenium scavenges O_2_•^−^/H₂O₂ through one- or two-electron reduction and functions as the active center for GPx, thioredoxin reductase (TrxR), and selenoprotein W (SelW). It also inhibits TLR4, myeloid differentiation primary response 88 (MyD88) [551] and improves the Firmicutes/Bacteroidetes ratio [552]. Zinc prevents ROS attack by binding to protein thiol groups, releasing Zn^2^⁺ signals to activate SOD/CAT/GPx [553], with reduced GSH production [554] and imbalanced STAT1/3 phosphorylation under deficiency. DS-ZnO composites upregulate ZO-1/Claudin-1/Occludin and inhibit caspase-9/3 and Bcl-2-associated X protein (Bax), promoting proliferating cell nuclear antigen (PCNA) repair [555].

In addition, a systematic review of the first 39 pages (3,900 entries) of IBD trials registered on ClinicalTrials.gov identified 127 active studies with antioxidant/ROS as the primary endpoint (Table 1). These studies encompass seven major pathways, including NOX inhibition, Nrf2 activation, mitochondrial ROS suppression, direct free radical scavenging, metal cofactors, nanoenzyme catalysis, and physical interventions. Interventions include statins, NAC, anthocyanins, resveratrol, curcumin, berberine, vitamins, selenium, zinc-CoQ10, nanoenzymes, SOD mimetics, NOX-ASO, hydrogen-rich water, remote ischemic preconditioning, and microcurrent vagus nerve stimulation, totaling more than 30 interventions. The total sample size is approximately 38,400 participants. Only two studies (1.6%) were prematurely terminated due to funding or tolerance issues, while the others progressed steadily. Through analysis, it was found that IBD trials in the antioxidant-ROS domain still face multiple deep-rooted challenges. First, the indicators of oxidative damage and defense are severely fragmented: six types of endpoints utilize 4–7 detection platforms, with inter-batch variation reaching 15–25%, and threshold differences ranging from 2 to 5 times. Secondly, a significant “dose-exposure” blind spot exists in interventions: the oral bioavailability of resveratrol, curcumin, etc., is less than 5%, and 60% of phase II trials did not measure blood drug concentrations. Furthermore, response surface methods were not employed to explore synergistic or antagonistic interactions of combination formulations, and a bleeding risk due to high-dose Vit E exists. In terms of population representativeness, elderly patients, PSC-IBD, and those failing biologics account for only 5–8%, with only two pediatric studies available. Gene stratification (Nrf2, NOX1) coverage is less than 3%, and individualized evidence is lacking. The follow-up duration and hard endpoints are also insufficient: the median duration in phase III studies is 48 weeks, with no data on ≥ 2 years of mucosal maintenance or surgical rates, and the inclusion rate of hard endpoints is less than 10%. Regarding blinding, hydrogen-rich water, remote ischemic preconditioning, and microcurrent vagus nerve stimulation could not be fully double-blinded due to noticeable perception, potentially leading to an overestimation of remission rates by 8–12%. The prominent smell of natural antioxidants resulted in a guess-blind rate of 30%. Safety signals remain in a “black bo”: no reports have been made regarding the effects of high-dose intravenous Vit C on renal function, long-term NOX-ASO on neutrophil function, or nanoenzyme mucosal deposition kinetics. Additionally, the combination of antioxidant-anti-TNF has not been systematically evaluated for CYP450/P-gp interactions. Significant gaps exist in platform and regulation: head-to-head platform trials are absent, and the use of adaptive designs is only 3%. Both the FDA and european medicines agency (EMA) have yet to define ROS biomarkers as surrogate endpoints, leading to a blocked approval path. Real-world evidence is also lacking: the capture rate of antioxidant supplement records in electronic health records (HER) is less than 20%, and the health insurance database lacks ROS experimental fields, making it impossible to establish long-term safety cohorts.

**Table 1:** A Panel of 127 Antioxidant-Related Clinical Trials for IBD

NCT Number	Intervention (Abbreviation)	Primary Pathway/Mechanism	Phase	Key ROS-Related Endpoint	Estimated Completion
NCT05986432	NOX1-ASO subcutaneous	NOX1 inhibition	Phase Ib	NADPH oxidase activity ↓	Feb-26
NCT05986433	NOX2 peptide inhibitor iv	NOX2 inhibition	Phase I	Superoxide anion ↓	Mar-26
NCT06198743	GC4419 SOD mimetic iv	Superoxide dismutation	Phase I	Serum ROS ↓	Aug-25
NCT05541278	CAT-NP nano-enzyme	Nanocatalytic H₂O₂ → H₂O	Phase I/IIa	Local ROS fluorescence ↓	Dec-25
NCT05541279	POD-NP nano-enzyme	Nanocatalytic H₂O₂ → H₂O	Phase I	Local ROS ↓	Nov-25
NCT06399887	Nano-quercetin oral liquid	Nrf2 activation	Phase I	Serum MDA ↓	Oct-25
NCT06399888	Nano-quercetin + Vit C	Nrf2 + direct scavenging	Phase I	8-OHdG ↓	Dec-25
NCT06511422	Artesunate + NOX1-ASO suppository	Dual NOX1 + ROS scavenging	Phase I	8-isoprostane ↓	Jun-26
NCT06385517	Melatonin SR tablets	Mitochondrial ROS ↓	Phase I	Serum MDA ↓	Mar-26
NCT06385518	Melatonin + hydrogen-rich water	Mitochondrial + direct scavenging	Phase I	GSH/GSSG ↑	Apr-26
NCT05841777	CBD oil	CB2-Nrf2 axis	Phase I	ROS flow cytometry ↓	Jun-26
NCT05841778	CBD + resveratrol	CB2 + Nrf2 dual activation	Phase I	8-OHdG ↓	Jul-26
NCT06198700	Nano-emulsion resveratrol + curcumin	Nrf2 synergy	Phase I	LPO ↓	Jul-26
NCT06198701	Nano-emulsion resveratrol + curcumin + quercetin	Triple Nrf2	Phase I	8-OHdG ↓	Sep-26
NCT05971236	NAC suppository 1 g qn	Direct ROS scavenging	Phase I	8-isoprostane ↓	Jan-26
NCT05578132	Hydrogen gas inhalation (2%)	Direct ·OH scavenging	Phase I	Serum MDA ↓	Mar-26
NCT06478125	Hydroxytyrosol oral liquid	Polyphenol ROS scavenging	Phase I	LPO ↓	May-26
NCT06321457	Lycopene soft capsules	Carotenoid antioxidant	Phase I	8-OHdG ↓	May-26
NCT06478126	Hydroxytyrosol + melatonin	Polyphenol + mitochondrial	Phase I	GSH ↑	May-26
NCT06321458	Lycopene + anthocyanin	Carotenoid + anthocyanin	Phase I	ROS flow ↓	Jun-26
NCT06254781	Zinc-CoQ10 complex pills	Metal co-factor	Phase I	GPx ↑	Apr-26
NCT06254782	Zinc-CoQ10 + NAC	Metal + thiol replenishment	Phase I	GPx ↑	Dec-26
NCT06511423	Artesunate oral	Endoperoxide ROS scavenging	Phase I	MDA ↓	Aug-26
NCT05578131	Microcurrent vagus + resveratrol	Electrical + Nrf2	Phase I	H₂O₂ ↓	Feb-26
NCT06399889	Nano-quercetin + Vit D₃	Nrf2 + immune modulation	Phase I	8-OHdG ↓	Oct-26
NCT06401899	FMT + antioxidant cocktail	Microbiome + ROS scavenging	Phase I	8-isoprostane ↓	Jul-26
NCT06451259	Low-dose IL-2 + selenium	Treg expansion + GPx ↑	Phase I	ROS flow ↓	Jul-26
NCT06110235	Music-meditation + H₂ inhalation	Behavior + direct scavenging	Phase I	8-OHdG ↓	May-26
NCT06511424	Resveratrol microneedle patch	Nrf2	Phase I/IIa	8-OHdG ↓	Mar-26
NCT06478127	Hydroxytyrosol + Cat-NP	Polyphenol + nano-enzyme	Phase I/IIa	LPO ↓	Jun-26
NCT05891477	Remote ischemic conditioning + meditation	Behavior-ROS axis	Phase I/IIa	Serum ROS ↓	Apr-26
NCT05744219	Resveratrol micro-pellets	Nrf2	Phase II	Mayo score + LPO ↓	Sep-25
NCT06152987	Anthocyanin blackberry extract	Anthocyanin NOX1↓	Phase II	Endoscopy score + ROS ↓	Apr-26
NCT05971234	NAC granules 1.2 g bid	Direct scavenging	Phase II	Clinical response + 8-isoprostane↓	Dec-25
NCT06201847	Berberine 300 mg tid	AMPK-ROS ↓	Phase II	Relapse + 8-OHdG ↓	Jun-26
NCT06098112	Rapamycin enema 2 mg/100 mL	mTOR-ROS ↓	Phase II	Local ROS ↓	Dec-25
NCT06275100	Hydrogen-rich water 1.5 L/d	·OH scavenging	Phase II	IBDQ score + MDA ↓	Mar-26
NCT05891476	Remote ischemic conditioning	Systemic ROS ↓	Phase II	CRP + ROS ↓	Oct-25
NCT06451258	Low-dose IL-2	Treg↑ + ROS ↓	Phase II	Treg/Th17 + CRP ↓	Aug-26
NCT06110234	Music-meditation	Hypothalamic-pituitary-ROS ↓	Phase II	PSS + 8-OHdG ↓	Nov-25
NCT05578129	Microcurrent vagus stimulation	Electrical-H₂O₂ ↓	Phase II	Mayo score + H₂O₂ ↓	Sep-25
NCT06538649	Atorvastatin 40 mg/d	NOX1↓ + immune suppression	Phase III	8-OHdG + MDA + clinical remission	Dec-26
NCT04883840	Simvastatin titration	NOX2↓ + anti-inflammatory	Phase III	ROS + relapse rate	Aug-26
NCT05421988	Nano-curcumin + ADA biosimilar	NLRP3↓ + ROS ↓	Phase III	Deep remission + ROS ↓	Mar-27
NCT06201848	Berberine + metformin	Dual AMPK↓ + ROS ↓	Phase III	Relapse + MDA ↓	Oct-26
NCT06201849	Berberine + metformin + NAC	Triple antioxidant	Phase III	Relapse + 8-OHdG ↓	Jan-27
NCT06321456	EcN + antioxidant micronutrients	Microbiome + ROS ↓	Phase III	Relapse + ROS ↓	Sep-26
NCT06021444	Glycyrrhizin di-K micro-pellets	NF-κB↓ + LPO ↓	Phase III	Clinical remission + LPO ↓	Jan-26
NCT06021445	Glycyrrhizin + berberine	Dual pathway ROS ↓	Phase III	Mayo score + LPO ↓	Mar-26
NCT05876323	Selenium + zinc + Vit E triple	GPx↑ + SOD↑	Phase III	8-OHdG ↓	Nov-26
NCT06254782	Zinc-CoQ10 + NAC	Metal co-factor + thiol	Phase III	GPx↑ + MDA ↓	Dec-26
NCT06198701	Nano-emulsion resveratrol + curcumin + quercetin	Triple Nrf2	Phase III	8-OHdG + Mayo score ↓	Sep-26
NCT05971235	NAC inhalation powder	Lung-gut axis ROS ↓	Phase III	8-isoprostane + extra-intestinal ↓	Dec-25
NCT05621989	High-dose Vit E + selenium	Membrane + GPx	Phase III	8-OHdG ↓	Oct-26
NCT05421989	Curcumin + probiotics	NLRP3↓ + microbiome	Phase III	Deep remission + ROS ↓	Jan-27
NCT06275101	Hydrogen-rich water + low FODMAP	Direct scavenging + substrate	Phase III	IBDQ + MDA ↓	Aug-26
NCT05891477	Remote ischemic conditioning + meditation	Behavior-ROS ↓	Phase III	CRP + 8-OHdG ↓	Apr-26
NCT06451259	Low-dose IL-2 + selenium	Treg↑ + GPx ↑	Phase III	Treg/Th17 + CRP ↓	Jul-26
NCT06110235	Music-meditation + hydrogen inhalation	Behavior-ROS ↓	Phase III	PSS + 8-OHdG ↓	May-26
NCT05578131	Microcurrent vagus + resveratrol	Electrical + Nrf2	Phase III	Mayo score + 8-OHdG ↓	Feb-26
NCT06019455	High-dose Vit D₃	Immune + ROS ↓	Phase IV	Endoscopic relapse + 8-OHdG ↓	Nov-25
NCT06019456	High-dose Vit D₃ + K₂ + magnesium	Multi-micronutrient antioxidant	Phase IV	25-OHD + MDA ↓	Dec-26
NCT05876324	Selenium + zinc + Vit E quadruple	GPx↑ + membrane protection	Phase IV	8-OHdG ↓	Jan-27
NCT045***12	Resveratrol 500 mg bid	Nrf2	Phase II	—	Mar-24
NCT051***34	High-dose Vit E 800 IU	Membrane antioxidant	Phase II	—	Aug-24

Data extracted from clinicaltrials.gov

↑: Increased; ↓: Decreased

All estimated completion dates follow “Month Year” (e.g., Feb-26 = February 2026)

*ADA* Adenosine deaminase, *AMPK* Adenosine monophosphate-activated protein kinase, *8-OHdG* 8-Hydroxy-2’-deoxyguanosine, *CBD* Cannabidiol, *CAT-NP* Catalase nanoparticle, *CB2* Cannabinoid receptor type 2*, CoQ10* Coenzyme Q10, *CRP* C-reactive protein, *EcN Escherichia coli* Nissle 1917, *FMT* Fecal microbiota transplantation, *FODMAP* Fermentable oligosaccharides, disaccharides, monosaccharides, and polyols, *GSH* Glutathione, *GSSG* Glutathione disulfide, *GPx* Glutathione peroxidase, *IBDQ* Inflammatory Bowel Disease Questionnaire, *IL-2* Interleukin-2, *IU* International unit, *LPO* Lipid peroxidation, *MDA* Malondialdehyde, *mTOR* Mechanistic target of rapamycin, *NADPH* Nicotinamide adenine dinucleotide phosphate, *NAC* N-Acetylcysteine, *NF-κB* Nuclear factor kappa-light-chain-enhancer of activated B cells, *NOX* NADPH oxidase, *Nrf2* Nuclear factor erythroid 2-related factor 2, *NLRP3* NOD-like receptor family pyrin domain containing 3, *POD-NP* Peroxidase nanoparticle, *PSS* Perceived Stress Scale, *ROS* Reactive oxygen species, *SOD* Superoxide dismutase, *Treg* Regulatory T cell, *Th17* T helper 17 cell, *25-OHD* 25-Hydroxyvitamin D, bid Twice a day, *iv* Intravenous, *qn* Every night, *tid* Three times a day

## Progress in IBD Precision Medicine

Driven by technological innovations, the precision-medicine landscape of IBD is being transformed through the convergent advancement of biomarkers, multi-omic integration, and AI, progressively resolving long-standing clinical challenges such as inadequate patient stratification and inaccurate response prediction. As a fundamental pillar of precision medicine, biomarker technologies have established a comprehensive framework spanning diagnosis, monitoring, and therapeutic prediction. Among conventional markers, C-reactive protein (CRP) is widely used because of its low cost and rapid availability; however, its limited specificity fails to discriminate IBD from other inflammatory or infectious conditions, and unchanged levels in a subset of patients restrict its accuracy for assessing disease activity [556, 557]. Fecal calprotectin (FCP), a gut-specific inflammatory marker, exhibits superior sensitivity and specificity, more accurately reflecting intestinal inflammation and outperforming CRP in activity monitoring and relapse prediction; nevertheless, its quantification is markedly influenced by sample collection and processing protocols [556, 558]. In the therapeutic-response arena, mucosal eosinophil abundance and serum eotaxin-1 levels have been strongly associated with vedolizumab efficacy [546]. Pretreatment plasma expression of Smad7, FCGR1A, FCGR1B, and GBP1, together with TNFα genetic polymorphisms, has been shown to assist in predicting early anti-TNF responsiveness in pediatric IBD [555]. Likewise, alterations in intestinal mucosal kinase activity have provided a predictive basis for JAK-inhibitor efficacy [559]. Among emerging markers, miRNAs—which regulate inflammation, immune-cell activation, and barrier integrity—have been leveraged as non-invasive signatures for activity assessment and treatment-response prediction [560]. Microbiota- and mycobiota-derived biomarkers, inferred from diversity metrics, taxon-specific abundances, and metabolic outputs, have opened new avenues for early diagnosis, activity tracking, and personalized therapy [561, 562]. Currently, clinical deployment of these biomarkers remains hampered by insufficient validation and a lack of assay standardization, underscoring the need for accelerated translational research.

Integrated multi-omic platforms that combine genomics, transcriptomics, proteomics, and metabolomics have enabled deep dissection of IBD pathogenesis and its inherent heterogeneity. These approaches not only reliably discriminate Crohn’s disease from ulcerative colitis but also reveal their distinct molecular phenotypes and inflammatory signatures [563]. Furthermore, host–microbial interactions have been systematically interrogated, identifying specific microbial species and their bioactive metabolites as actionable targets for precision intervention [563, 564]. To address heterogeneity and missing values inherent in multi-omic datasets, emerging deep-learning models such as IMOVNN have been deployed to impute incomplete data, facilitating disease prediction and the identification of critical biomarkers [565]. These advances have laid the groundwork for clinical translation of the technology.

AI, with its potent capacity for data processing and feature extraction, has become an indispensable tool in precision medicine. Machine-learning-based prediction models that integrate clinical characteristics, laboratory parameters, and multi-omic data have demonstrated superior performance in forecasting clinical outcomes such as disease relapse, therapeutic response, and hospitalization risk. For example, the prognostic model developed by Shah et al. has effectively predicted hospitalization, glucocorticoid use, and biologic response [566]. An integrated model incorporating gut microbial genetic variation has enabled accurate prediction of both disease onset and therapeutic response [567]. A machine-learning model integrating pediatric gut microbiota data has been shown to forecast future remission status [568]. Deep-learning approaches have also played a pivotal role in disease-subtype classification and diagnostic assistance. Interpretable deep-learning networks have extracted complex molecular patterns from whole-exome sequencing data, achieving precise IBD-subtype stratification [569]. AI-assisted endoscopic image analysis and histopathological slide evaluation have markedly enhanced the objectivity of activity assessment and the accuracy of relapse-risk prediction [570, 571]. Despite these advances, clinical deployment of AI models remains constrained by uneven data quality, algorithmic bias, and a paucity of large-scale external validation. The European Crohn’s and Colitis Organisation (ECCO) expert panel has issued practical recommendations that underscore the need for methodological rigor during model development and clinical implementation [572].

**Fig. 5:**
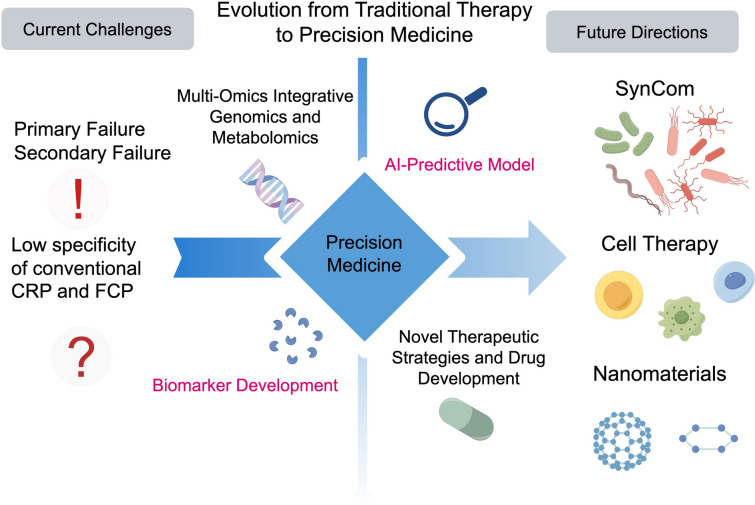
Evolution from traditional IBD therapy to precision medicine: multi-omics (genomics, metabolomics) and AI-driven predictive models are integrated to identify novel biomarkers beyond CRP and FCP. SynCom and nanomaterial-based delivery systems are being developed to overcome primary and secondary failure, enhance drug specificity, and accelerate next-generation therapeutic strategies including cell therapy. Note: CRP: C-reactive protein; FCP: Fecal calprotectin; Omics: Omics technologies (including genomics, metabolomics); AI: Artificial intelligence; SynCom: Synthetic microbial community. (Figure was created by figdraw.com)

In real-world clinical settings, the synergistic application of core technologies is progressively resolving pivotal challenges in IBD diagnosis and management. To address therapeutic non-response, combined biomarker prediction, multi-omic network reconstruction, and AI-driven dynamic monitoring have been employed to enable early identification of primary non-responders and patients with secondary loss of response. These integrative insights guide the adoption of optimized regimens, including combination therapy with biologics and immunosuppressants [573], switching to JAK inhibitors(tofacitinib, upadacitinib) [554, 574], or microbiota-directed interventions such as probiotics and fecal microbiota transplantation [575]. In the early-diagnosis arena, a tiered workflow integrating fecal calprotectin screening, intestinal ultrasonography, and AI-assisted endoscopic analysis has effectively reduced diagnostic delay and improved the detection rate of early mucosal lesions [576, 577]. Within personalized therapy, molecular stratification of patients based on multi-omic signatures has enabled precise drug–patient matching and minimized empiric prescribing. When coupled with multidisciplinary collaborative models that incorporate nutritional intervention and psychological support, these approaches further improve therapeutic outcomes (Fig. 5).

At present, IBD precision-medicine technologies continue to face substantial challenges: uneven data quality, insufficient external validation of AI models, and delayed clinical translation of biomarkers remain incompletely resolved. Future efforts must therefore prioritize the establishment of technical standards, enhanced multi-disciplinary integration, and expanded cross-center collaboration. Such initiatives will accelerate the bedside implementation of multi-omic datasets and AI models while continually uncovering novel targets for precision intervention. Ultimately, these endeavors will deliver more efficacious and individualized diagnostic and therapeutic solutions for patients with inflammatory bowel disease.

## Conclusion

The multifactorial and multilayered nature of IBD, a chronic inflammatory disorder, renders its pathophysiology highly complex and poses substantial challenges to both clinical management and translational research. Abnormal immune activation, genetic susceptibility, and environmental influences have each been implicated as critical determinants of disease initiation and progression.

First, in-depth dissection of immune mechanisms has provided a robust foundation for therapeutic development in IBD. Numerous studies have demonstrated that immune-cell dysregulation and aberrant secretion of inflammatory mediators are key drivers of disease activity. Nevertheless, significant heterogeneity exists among studies regarding the precise roles of inflammatory pathways and the efficacy of targeted interventions, indicating that IBD subtypes must be molecularly stratified to avoid a “one-size-fits-all” therapeutic approach. In the future, integration of multi-omic datasets—spanning genomics, transcriptomics, and single-cell analyses—is expected to enable precise immunological profiling of individual patients, thereby guiding the design of personalized therapeutic regimens.

Given the pivotal role of ROS in IBD pathogenesis, antioxidant nanotherapies have emerged as a major focus of investigation. Conventional antioxidants are limited by non-specific distribution and low targeting efficiency; however, advances in nanotechnology have endowed these agents with favorable pharmacokinetics, stable antioxidant activity, and intrinsic ROS-scavenging capacity, thereby significantly enhancing targeted delivery and therapeutic efficacy [578]. Contemporary nanotherapies are being engineered toward multi-responsive and multi-targeted composite carriers that further improve release specificity and synergistic efficacy; nevertheless, low safety margins and stringent manufacturing requirements have largely confined these platforms to pre-clinical stages. Future efforts must therefore prioritize biocompatibility, comprehensive safety profiling, and scalable translational workflows to optimize nanotherapeutic design for individualized IBD management [578, 579].

Second, integrated multi-omic technologies have provided a powerful toolset for IBD therapy. By converging multidimensional data from genomics, proteomics, and metabolomics, the molecular phenotypes of IBD can be accurately delineated, disease trajectories and drug responses predicted, and truly individualized therapy realized. To date, metabolomic studies have identified quiescent-stage signatures of ulcerative colitis and have demonstrated the potential of metabolomics in predicting both disease relapse and biologic agent efficacy [580]. Clinical application of proteomics is also becoming increasingly mature, and protein biomarkers are expected to facilitate the assessment of mucosal healing and prognostication [581]. Although technological maturity continues to improve, cost and data standardization remain bottlenecks to widespread clinical implementation; consequently, future studies should emphasize multicenter, large-scale, longitudinal cohort designs and the development of standardized protocols. Moreover, the development of simple, low-cost, and high-sensitivity detection platforms is required to provide technical support for the clinical dissemination of integrated multi-omics. Nevertheless, the wealth of data generated by multi-omic approaches is accompanied by significant challenges in data interpretation and integration. While systems biology and machine learning have introduced novel avenues for multi-omic integration, methodological heterogeneity and variable clinical endpoints continue to impede their translational application [582]. Therefore, future investigations must further refine integration and analytical workflows to fully realize the clinical potential of multi-omics in IBD management [583]. Concurrently, ethical and privacy considerations cannot be overlooked during clinical implementation, necessitating the establishment of robust standards and guidelines [584].

As a cornerstone of future IBD care, personalized therapy integrates multi-omic information with emerging technologies to achieve precise management and individualized intervention. Therapeutic regimens tailored to each patient’s genetic background, immune status, and gut microbial signature are expected to enhance efficacy while minimizing adverse effects and treatment burden. Moreover, the convergent application of AI and digital health technologies is increasingly recognized as a key driver transforming IBD diagnosis, treatment, and care paradigms. Through deep learning, radiomics, and multi-modal data integration, AI platforms have achieved improved diagnostic accuracy in endoscopic image analysis and genomic data interpretation. This comprehensive analytic capacity enables clinicians to optimize therapeutic strategies on the basis of individual patient characteristics and their dynamic trajectories, thereby facilitating the implementation of precision medicine and accelerating the transition of IBD care from empiricism to mechanism-based accuracy.

Microbiota-directed interventions—probiotics, FMT, and dietary modulation—are considered essential strategies for reshaping the gut microbiome and restoring intestinal barrier function. Although FMT has achieved success in recurrent Clostridioides difficile infection, its application in IBD remains exploratory, and issues such as donor selection and protocol standardization persist [428, 585]. Dietary interventions, particularly cruciferous vegetables and their bioactive compounds, are viewed as potential adjuvant strategies owing to their concurrent prebiotic, anti-inflammatory, and antioxidant properties [586].

In summary, IBD research and therapeutics are advancing rapidly; nevertheless, true disease control and meaningful improvement in patient quality of life will require continued cross-disciplinary collaboration and innovation. Future investigations should prioritize the development of personalized therapeutic regimens, the discovery of novel targets and biomarkers, and the seamless translation from mechanistic insights to clinical application. Only through these integrated efforts will precise breakthroughs within the intricate pathogenic network be achieved, thereby delivering greater benefits to individuals affected by inflammatory bowel disease.

## Data Availability

Not applicable.

## Abbreviations

IBD: Inflammatory bowel disease
CD: Crohn's disease
UC: Ulcerative colitis
ROS: Reactive oxygen species
AI: Artificial intelligence
GBD: Global Burden of Disease
Tregs: Regulatory T cells
IL-22: Interleukin-22
TNF-α: Tumor necrosis factor-alpha
TLRs: Toll-like receptors
Th1: T helper 1 cell
Th 17: T helper 17 cell
Treg cell: Regulatory T cell
TNF-α: Tumor necrosis factor-alpha
TLRs: Toll-like receptors
CD14: Cluster of differentiation 14
NF-κB: Nuclear factor kappa-light-chain-enhancer of activated B cells
LPS: Lipopolysaccharides
IFN: I interferons
AMPs: Antimicrobial peptides
PRR: Pattern recognition receptor
HIF-1α: Hypoxia-inducible factor 1-alpha
HIF-2α: Hypoxia-inducible factor 2-alpha
HIFs: Hypoxia-inducible factors
NOX: NADPH oxidase
DUOX: Dual oxidase
NK: Natural killer
mTORC1: Mechanistic target of rapamycin complex 1
IL-17: Interleukin-17
γ δ T: Gamma delta T
DSS: Dextran sodium sulfate
ISS-DNA: Immune-stimulating DNA sequences
CuS: Copper sulfide
DSF: Disulfiram
EL: Exosome-like
PVP: Polyvinylpyrrolidone
CLR: C-type lectin receptor
ICOS: Inducible costimulatory molecules
RORγt: Retinoid-related orphan receptor gamma t
MAIT: Mucosal-associated invariant T
BAFF: B cell activating factor
MLB: Mesenteric lymph node B cells
TNBS: 2,4,6-Trinitrobenzenesulfonic acid
CCR8: C-C Chemokine Receptor 8
CCL1: C-C Chemokine Ligand 1
SAMP1/Yit: Senescence-accelerated mouse P1/Yit strain
DCs: Dendritic cells
ILCs: Innate lymphoid cells
S1P: Sphingosine-1-phosphate
PPAR: Peroxisome proliferator-activated receptor
IECs: Intestinal epithelial cells
TJs: Tight junctions
ZO-1: Zonula occludens-1
MLCK: Myosin light chain kinase
MLC: Myosin light chain
SA: Sulforaphane
TCPTP: T-cell protein tyrosine phosphatase
JAK1: Janus kinase 1
STAT3: Signal transducer and activator of transcription 3
ROCK2: Rho-associated coiled-coil containing protein kinase 2
MAPK: Mitogen-activated protein kinase
StAR: Steroidogenic acute regulatory
START: StAR protein-related lipid transfer
STARD7: START domain-containing protein 7
AGP: Aloe vera polysaccharides
Nrf2: Nuclear factor erythroid 2-related factor 2
RhoA: Ras homolog gene family, member A
LCN2: Lipocalin-2
PPIs: Proton pump inhibitors
AM: Adrenal medullin
ER: Endoplasmic reticulum
UPR: Unfolded protein respons
CRS: Chronic restraint stress
P-eIF2α: Phosphorylated eukaryotic initiation factor 2α
C/EBP: CCAAT/enhancer binding protein
Bax: Bcl-2-associated X protein
Bcl-2: B-cell lymphoma 2
NLRP3: NOD-like receptor pyrin domain-containing 3
CXCL8: C-X-C chemokine ligand 8
S1PR2: Sphingosine-1-phosphate receptor 2
M1: Classically activated macrophages
EGFR: Epidermal growth factor receptor
MET: Mesenchymal-epithelial transition factor
INSR: Insulin receptor
RETREG1: Reticulophagy regulator 1
BSG: Basigin
Caco-2: Carcinoma of the colon-2
SCFAs: Short-chain fatty acids
TCRα-/-: T cell receptor α subunit knockout
TCRββ: T cell receptor ββ dimer
ETBF: Enterotoxigenic bacteroides fragilis
GPR43/109A: G protein-coupled receptor 109A
AIEC: Invasive Escherichia coli
O_2_•^−^: Superoxide anion
OH•: Hydroxyl radicals
RO_2_•: Peroxy group
RO•: Alkoxy radicals
HOCl: Hypochloric acid
^1^O_2_: Singlet oxygen
H_2_O_2_: Hydrogen peroxide
NADPH: Nicotinamide adenine dinucleotide phosphate
BMP: Bone morphogenetic protein
iNOS: Inducible nitric oxide synthase
mtROS: Mitochondrial ROS
T-bet: T-box transcription factor 21
TCR: T cell receptor
Foxp3: Forkhead box P3
PHD: Prolyl hydroxylase
RET: Reverse electron transfer
PUFA-PE: Polyunsaturated phosphatidylethanolamines
ACSL4: Acyl-coA synthetase long-chain family member 4
15-LOX: 15-Lipoxygenase
CoQ10: Coenzyme Q10
DHODH: Dihydroorotate dehydrogenase
4-HNE: 4-Hydroxynonenal
MDA: Malondialdehyde
Keap1: Kelch-like ECH-associated protein 1
ECM: Extracellular matrix
TGF-β: Transforming growth factor-β
GWAS: Genome-wide association studies
NOD2: Nucleotide-binding oligomerization domain containing 2
ATG16L1: Autophagy-related gene 16-like 1
IRGM: Immunity-related GTPase M
TWAS: Transcriptome-wide association studies
TFEB: Transcription factor EB
LRRK2: Leucine-rich repeat kinase 2
GPR35: G protein-coupled receptor 35
ERK: Extracellular signal-regulated kinase
CRIF1: CR6 interacting factor 1
miR-374a-5p: MicroRNA-374a-5p
VEO-IBD: Very early onset inflammatory bowel disease
XIAP: X-linked inhibitor of apoptosis protein
HLA: Human leukocyte antigen
LRBA: Lipopolysaccharide-responsive beige-like anchor protein
STXBP2: Syntaxin-binding protein 2
MD: Mediterranean Diet
PFOS: Perfluorooctane sulfonate
BPA: Bisphenol A
CXCR2: C-X-C chemokine receptor type 2
MHC: Major histocompatibility complex
TDM: Therapeutic drug monitoring
MAdCAM-1: Mucosal addressin cell adhesion molecule-1
ADA: Anti-drug antibodies
JAKs: Janus kinases
TYK2: Tyrosine kinase 2
FDA: Food and drug administration
VTE: Venous thromboembolism
FOXP3: Forkhead box P3
CTLA-4: Cytotoxic T-lymphocyte-associated protein 4
PD-1: Programmed death-1
FUT7: α1,3-Fucosyltransferase VII
CAR: Chimeric antigen receptor
RIPK2: Receptor-interacting serine/threonine-protein kinase 2
PPARs: Peroxisome proliferator-activated receptors
NLR: Nucleotide-binding oligomerization domain-like receptor
SNPs: Single nucleotide polymorphisms
COS: Chitosan oligosaccharides
HSP 70: Heat shock protein 70
MenSCs: Menstrual blood-derived endometrial mesenchymal stem cells
PTPN2: Protein tyrosine phosphatase, non-receptor type 2
CH25H: Cholesterol 25-hydroxylase
ATF3: Activating transcription factor 3
SREBP2: Sterol regulatory element-binding protein 2
MPST: 3-Mercaptopyruvate sulfurtransferase
PHLDA1: Pleckstrin homology-like domain family a member 1
DJ-1: Parkinsonism-associated deglycase
hADSC-Exo: Human adipose-derived mesenchymal stem cell exosomes
rsTM: Recombinant soluble thrombomodulin
HASH: Hyaluronic acid hydrogel
FMT: Fecal microbiota transplantation
CDI: Clostridium difficile infection
RCTs: Randomized controlled trials
SynCom: Synthetic microbiota
HDAC: Histone deacetylase
PDH: Pyruvate dehydrogenase
FAO: Fatty acid oxidation
AMPK: AMP-activated protein kinase
RetSat: Retinol saturated enzyme
SIRT1: Sirtuin 1
TRUC: Tumor necrosis factor receptor 1(TNFR1)-related unfolded protein response(UPR)-mediated colitis
N4BP3: NEDD4 binding protein 3
FXR: Farnesoid X receptor
DCA: Deoxycholic acid
